# Ovarian cancer‐associated mesothelial cells induce acquired platinum‐resistance in peritoneal metastasis *via* the FN1/Akt signaling pathway

**DOI:** 10.1002/ijc.32854

**Published:** 2020-01-24

**Authors:** Masato Yoshihara, Hiroaki Kajiyama, Akira Yokoi, Mai Sugiyama, Yoshihiro Koya, Yoshihiko Yamakita, Wenting Liu, Kae Nakamura, Yoshinori Moriyama, Hiroaki Yasui, Shiro Suzuki, Yusuke Yamamoto, Carmela Ricciardelli, Akihiro Nawa, Kiyosumi Shibata, Fumitaka Kikkawa

**Affiliations:** ^1^ Department of Obstetrics and Gynecology Nagoya University Graduate School of Medicine Nagoya Japan; ^2^ Bell Research Center, Department of Obstetrics and Gynecology Collaborative Research Nagoya University Graduate School of Medicine Nagoya Japan; ^3^ Bell Research Center for Reproductive Health and Cancer Nagoya Japan; ^4^ Division of Molecular and Cellular Medicine National Cancer Center Research Institute Tokyo Japan; ^5^ Discipline of Obstetrics and Gynaecology Adelaide Medical School, Robinson Research Institute, University of Adelaide Adelaide SA Australia; ^6^ Department of Obstetrics and Gynecology Fujita Health University Bantane Hospital Nagoya Japan

**Keywords:** FN1/Akt signaling, Mesothelial cell, ovarian cancer, peritoneal dissemination, platinum drug resistance

## Abstract

Peritoneal dissemination of ovarian cancer (OvCa) arises from the surface of the peritoneum, covered by monolayer of mesothelial cells (MCs). Given that both OvCa cells and MCs are present in the same peritoneal metastatic microenvironment, they may establish cell‐to‐cell crosstalk or phenotypic alterations including the acquisition of platinum‐resistance in OvCa cells. Herein, we report how OvCa‐associated mesothelial cells (OCAMs) induce platinum‐resistance in OvCa cells through direct cell‐to‐cell crosstalk. We evaluated mutual associations between OvCa cells and human primary MCs with *in vitro* coculturing experimental models and *in silico* omics data analysis. The role of OCAMs was also investigated using clinical samples and *in vivo* mice models. Results of *in vitro* experiments show that mesenchymal transition is induced in OCAMs primarily by TGF‐β1 stimulation. Furthermore, OCAMs influence the behavior of OvCa cells as a component of the tumor microenvironment of peritoneal metastasis. Mechanistically, OCAMs can induce decreased platinum‐sensitivity in OvCa cells *via* induction of the FN1/Akt signaling pathway *via* cell‐to‐cell interactions. Histological analysis of OvCa peritoneal metastasis also illustrated FN1 expression in stromal cells that are supposed to originate from MCs. Further, we also confirmed the activation of Akt signaling in OvCa cells in contact with TGF‐β1 stimulated peritoneum, using an *in vivo* mice model. Our results suggest that the tumor microenvironment, enhanced by direct cell‐to‐cell crosstalk between OvCa cells and OCAMs, induces acquisition of platinum‐resistance in OvCa cells, which may serve as a novel therapeutic target for prevention of OvCa peritoneal dissemination.

## What's new?

The clinical characteristics of refractory advanced ovarian cancer suggest that the peritoneum acts as an anchoring point for metastatic tumors, enabling persistent ovarian cancer (OvCa) cell survival. This study shows that ovarian cancer‐associated mesothelial cells (OCAMs) and OvCa cells invade the extracellular matrix of the peritoneum. OCAMs were further found to directly influence persistent OvCa cell survival *via* FN1 signaling. FN1 on the surface of OCAMs induced activation of the Akt signaling pathway, thereby fueling platinum resistance in OvCa cells. The findings highlight the potential for targeting OCAMs as a novel therapeutic strategy for preventing peritoneal dissemination of ovarian cancer.

**Abbreviations**
CAF: cancer‐associated fibroblasts
DAVID: the database for annotation, visualization and integrated discovery
DEG: differentially expressed gene
ECM: extracellular matrix
EMT: epithelial–mesenchymal transition
FACS: fluorescence‐activated cell sorting
FBS: fetal bovine serum
GSEA: gene set enrichment analysis
HPMC: human peritoneal mesothelial cell
KEGG: Kyoto encyclopedia of genes and genomes
LC–MS/MS: liquid chromatography‐mass spectrometry
MC: mesothelial cell
OCAM: ovarian cancer‐associated mesothelial cell
OvCa: ovarian cancer
RI: TGF‐β1 receptor inhibitor
TCGA: the cancer genome atlas
TGF‐β1: transforming growth factor‐beta 1
UCSC: the university of California, Santa Cruz

## Introduction

Ovarian cancer (OvCa) is one of the leading causes of death among gynecological malignancies.^1^, ^2^ More than half of the patients with OvCa are diagnosed at an advanced stage due to lack of specific symptoms and effective early detection screening methods.^3^, ^4^, ^5^ Peritoneal dissemination, which is one of the most common causes of metastasis in the abdominal cavity, is frequently observed in patients with advanced OvCa.^6^, ^7^ Even when metastatic tumors are optimally resected, persistent cancerous cells often emerge to form new tumors despite the use of conventional platinum‐based chemotherapy.^8^, ^9^ This is the fundamental reason for the observed poor prognoses in patients with therapy‐resistant OvCa, an issue that has not improved significantly over the past few decades.^10^

The overall efficacy of the platinum anticancer agents ultimately determines the survival outcome in patients with advanced OvCa. Treatment‐free intervals act as a clinically significant prognostic factor for the recurrence of disease.^11^, ^12^ A variety of mechanisms have been reported to be associated with the development of platinum‐resistant cancer cells, including reduced membrane transport of the drug, and increased DNA repair mechanisms.^13^ Despite these molecular cell‐based findings, a novel therapeutic breakthrough for platinum‐resistant OvCa has yet to be developed. We are, therefore, required to look beyond molecular mechanisms specific to cells of a given carcinoma, and instead broaden our investigation to include the surrounding tumor microenvironment, which may provide important information regarding the global systemic mechanisms associated with resistance to platinum drugs. Moreover, the identification of general molecular targets will serve to inform the development of effective universal therapeutic options to prevent peritoneal dissemination of OvCa.

Peritoneal dissemination of OvCa is presumed to arise from the spread of cancer cells *via* the ascites.^14^ Furthermore, the surface of the peritoneum is histologically covered by a single layer of mesothelial cells (MCs),^15^ which may have a key function in the development of the tumor microenvironment that supports peritoneal metastasis of OvCa. Moreover, recent studies have shown that activated MCs play an important role in the development of peritoneal metastasis;^16^, ^17^, ^18^, ^19^, ^20^, ^21^, ^22^ these studies further demonstrated that MCs increase the adhesive and proliferative properties of OvCa cells. In fact, mesenchymal transition of MCs was reported to be induced by a variety of soluble factors in malignant ascites,^23^ and modification of extracellular matrix (ECM) on the mesothelial cells also promoted peritoneal metastasis of OvCa.^24^ These findings suggest that MCs no longer function as simply passive bystanders, but rather act as coordinators for the progression of OvCa. Additionally, cancer‐associated fibroblasts (CAFs) are recognized as a key component in the tumor microenvironment, and are reported to originate from various types of cells,^25^ including MCs, which have been described as a potential source of CAFs in peritoneal metastasis of OvCa, specifically. Given that both OvCa cells and MCs are present in the same peritoneal metastatic microenvironment, it may, therefore, be possible to establish cell‐to‐cell crosstalk or phenotypic alterations including the acquisition of platinum‐resistance in OvCa cells. However, to date, few studies have examined the direct interactions between these two cell types.

Herein, we report that OvCa‐associated mesothelial cells (OCAMs) promote the progression of advanced OvCa. With novel insights into the development of peritoneal metastasis, we investigated how OCAMs alter OvCa cells through direct cell‐to‐cell crosstalk. We also identified a key signaling pathway associated with the development of OCAM‐induced platinum‐resistance in OvCa cells. These findings serve to elucidate molecular mechanisms associated with a challenging clinical feature, namely, platinum‐resistant OvCa cells.

## Materials and Methods

### Ethical statement

Informed consent was obtained from patients prior to the collection of all biological samples according to the regulations set out by the Ethics Committee at Nagoya University. Our study including the animal experimental protocols were also approved by Nagoya University, and all experiments were conducted in accordance with the guidelines for animal experiments at Nagoya University.

### Cell cultures

ES‐2 (RRID:CVCL_3509), SKOV3 (RRID:CVCL_0532) and OV90 (RRID: CVCL_3768) cell lines were maintained in RPMI‐1640 media supplemented with 10% fetal bovine serum (FBS) and penicillin/streptomycin. All cell lines were obtained from ATCC (Manassas, VA) and were authenticated using short tandem repeat profiling (BEX, Tokyo, Japan) within the last 3 years. All experiments were performed with mycoplasma‐free cells. Stable cell lines expressing GFP were generated as described previously.^19^ Human peritoneal mesothelial cells (HPMCs) were isolated, as we have previously reported,^26^ from the tumor‐free omentum of patients with malignant ovarian tumors. The HPMCs were cultured on collagen‐coated plates in RPMI‐1640 media supplemented with 10% FBS and penicillin/streptomycin. HPMCs, in complete media, were treated with, or without transforming growth factor‐beta 1 (TGF‐β1; R&D Systems, Minneapolis, MN) in the presence or absence of 1.0 μmol/l of TGF‐β1 receptor inhibitor (RI), which inhibits the TGF‐β type I receptor, activin receptor‐like kinase 5^27^ (SB‐431452, R&D Systems) in RPMI‐1640 media supplemented with 10% FBS. We principally used HPMCs from different patients and repeated these experiments multiple times.

### Clinical ascites samples

Primary human ascites samples were collected from patients with OvCa. All samples were centrifuged at 1,500 rpm for 5 min and the supernatants were stored at −20°C.

### Apoptosis detection assay and fluorescence‐activated cell sorting analysis

HPMCs were plated on collagen‐coated six‐well plates and cultured until 100% confluence was achieved. After culturing with or without TGF‐β1 for 72 hr, GFP‐labeled OvCa cells were plated and cocultured with HPMCs for an additional 48 hr. Next, 1 × 10^5^ HPMCs were plated and cultured for 24–48 hr, followed by treatment with siRNAs for 24 hr and treated with 10 ng/ml of TGF‐β1. GFP‐labeled ES‐2 cells were plated and cocultured with the siRNA‐treated HPMCs for 48 hr.

To perform apoptosis detection assays, GFP‐labeled ES‐2 cells were incubated with cisplatin (30 μg/ml) for 24–48 hr. Alternatively, GFP‐labeled SKOV3 and OV90 cells were cultured in 10 and 50 μg/ml of cisplatin, respectively. When evaluating the impact of Akt, an Akt inhibitor (14,870, Cayman Chemical, 2 μmol/l) was added to the cultures 24 hr prior to addition of cisplatin. Apoptosis was analyzed using APC‐Annexin V with 7‐AAD staining according to the manufacturer's instructions (BD Biosciences, San Jose, CA). Fluorescence‐activated cell sorting (FACS, FACS Aria Cell Sorter, BD Biosciences) analysis was performed using GFP‐labeled ES‐2 cells that were sorted 48 hr after coculturing had begun, the protein and RNA were extracted for further analysis. The data were analyzed using FlowJo data analysis software package (BD Biosciences). Detailed sequences of the siRNAs used for the experiments are listed in Supporting Information Table S1.

### RNA microarray analysis

Detailed RNA microarray materials and methods are described in the Supporting Information. Gene expression analysis was performed using one‐way analysis of variance to identify differentially expressed genes (DEGs). Values of *p* and fold‐changes were calculated for each analysis. Unsupervised clustering and heat map generation were performed with sorted datasets using Pearson's correlation on Ward's method with selected Partek Genomics Suite 6.6. Gene set enrichment analysis (GSEA) (http://www.broadinstitute.org/gsea) probe sets were used to compare cocultured ES‐2 cells with untreated HPMCs and TGF‐β1‐treated HPMCs.

### Genetic analysis using The Cancer Genome Atlas database

Clinical records and RNA sequence data for patients with OvCa were obtained from The Cancer Genome Atlas (TCGA) on the University of California, Santa Cruz (UCSC) cancer genome browser (http://xena.ucsc.edu). Patients with valid expression data (n = 149) for the 12 genes of interest (*MXRA5*, *MMP2*, *FAP*, *COL1A2*, *COL1A1*, *THBS2*, *VAV3*, *FBN1*, *ADCY2*, *CXCL6*, *TNFRSF11B* and *MATN2*) were selected along with the corresponding criteria. Within the cohort, based on the distribution of each gene expression value, one‐third of patients (*n* = 50) with the highest expression level of the most upregulated genes, as determined *via* microarray analysis (MXRA5, MMP2, FAP, COL1A2, COL1A1, THBS2, VAV3, FBN1 and ADCY2), were assigned one point. Similarly, one‐third of the patients with the lowest expression of downregulated genes (*CXCL6*, *TNFRSF11B* and *MATN2*) were also assigned one point. The cumulated points for each patient were recognized by a mathematical scoring model that reflected the degree by which an individual patient's gene expression resembled that of ES‐2 cells cocultured with TGF‐β1‐treated HPMCs, or ES‐2 cells cocultured with untreated HPMCs. The patients were then divided into two groups (high scoring and low scoring). Differences relating to disease stage distribution, and progression‐free survival outcomes, were compared between the two groups.

### Proteomic analysis

Triplicate samples were prepared according to our previous report.^28^ We analyzed all proteins detected in each sample and every value determined to fall beneath the level of detection sensitivity was substituted by the adjusted minimum detection number. A fivefold change was set as the value for the geographic average between the paired groups as determined by the Mascot program, as the threshold for the protein abundance ratio to confidently identify proteins with significantly altered expression, as was previously reported.^29^ Using a bioinformatics web tool, the database for annotation, visualization and integrated discovery (DAVID, http://david.abcc.ncifcrf.gov, and version 6.8), we performed the enrichment of biological pathway analysis using Kyoto encyclopedia of genes and genomes (KEGG) with all the altered proteins. Subsequent Proteomaps of all identified annotated proteins in ES‐2 cells or HPMCs, were generated to visualize the differential contribution of biological pathways (http://bionic-vis.biologie.uni-greifswald.de/, version 2.0). Additionally, we used STRING, a bioinformatics web tool, (http://www.string-db.org/, version 10), to perform an interactome analysis on all of the altered proteins.

### *In vivo* studies

Control PBS or TGF‐β1 (20 ng/ml) were injected into the peritoneal cavity of 6‐ to 7‐week‐old female BALB/c nude mice (Japan SLC, Nagoya, Japan) once per day for a total of 5 days. Twenty‐four hours after the last injection, mice were sacrificed and protein from the peritoneum was extracted. For immunofluorescence analysis, the peritoneum was washed with PBS, fixed with PFA and stained with DAPI, phalloidin and an anti‐FN1 antibody. Additionally, ES‐2 cells were stained with CellTracker Green CMFDA dye, and 4 × 10^6^ cells were injected into the peritoneal cavity of the mice 24 hr after the last PBS or TGF‐β1 injection was administered. After an additional 24 hr, the mice were sacrificed, and the peritoneum was washed with PBS, fixed with PFA and stained with phalloidin as well as an anti‐phospho‐Akt antibody. The peritoneum was observed using a laser confocal microscope and photographs were obtained using IMARIS software (Bitplane, Switzerland). The images were then analyzed using the ImageJ software (NIH, Bethesda, MD). The procedure was carried out in precisely the same way for the observing, reconstructing and analyzing protocols.

### Statistical analysis

All data are presented as mean ± standard error. Statistical significance was analyzed using the Student's *t*‐test and chi‐square test as appropriate. The log‐rank test was used to assess differences in the survival trend between the groups, and hazard of recurrence was estimated using Cox regression analyses. A *p* < 0.05 denoted two‐sided statistical significance.

### Data availability

The data that supports the findings of our study are available upon request from the corresponding author. The data are not publicly available due to privacy or ethical restrictions.

## Results

### OCAMs interact with OvCa cells to create a permissive tumor microenvironment

**Figure 1:**
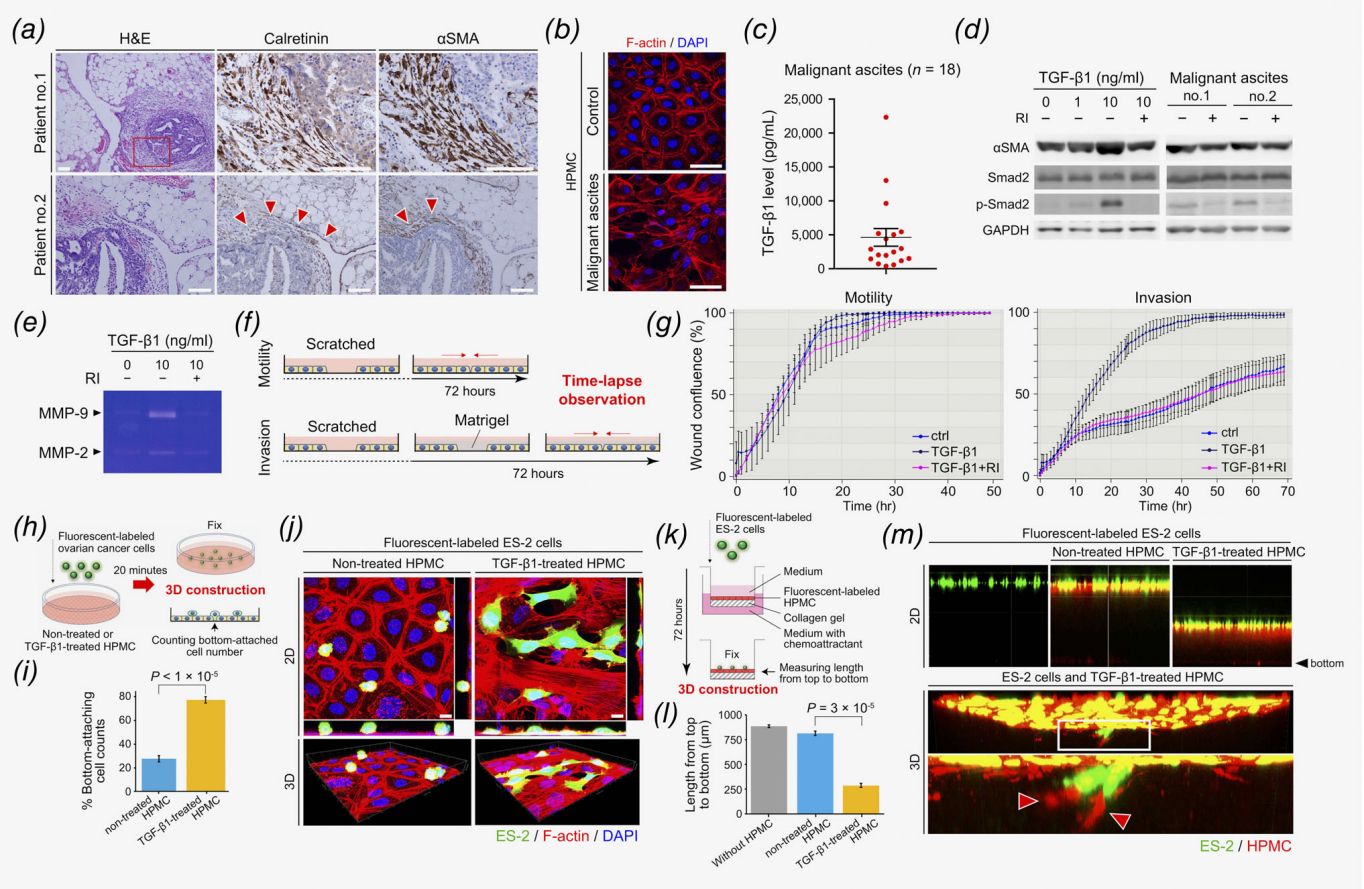
OCAMs contribute to the tumor microenvironment of peritoneal dissemination of OvCa. (*a*) H&E staining and immunohistochemistry of calretinin and αSMA in peritoneal dissemination of OvCa. Red boxed area in the left upper panel indicates the field observed in the right upper two panels. Arrowheads represent calretinin‐ and αSMA‐positive fibroblastic cells surrounding tumor cells, which were observed to be associated with the MC monolayer of the peritoneum. Scale bars, 100 μm. (*b*) Image of HPMCs cultured with control media or malignant OvCa ascites, stained with phalloidin and DAPI. Scale bars, 50 μm. (*c*) Concentration of TGF‐β1 detected in malignant ascites of OvCa. (*d*) Immunoblot analysis of HPMCs treated with TGF‐β1 and of malignant ascites cultured with or without RI. (*e*) Gelatin zymography of supernatant from HPMCs stimulated with TGF‐β1 and/or RI. (*f*) Schematic depicting the time‐lapse scratch assay used to assess the motility and invasiveness of HPMCs. (*g*) Wound confluence percentages of HPMCs stimulated with TGF‐β1 and/or RI were analyzed and plotted every hour. (*h*) Schematic protocol for trans‐mesothelial migration assay. (*i*) Number of ES‐2 cells attached to the glass identified in coculture with HPMCs either treated with control media or media containing TGF‐β1. (*j*) Representative 2D and 3D images of cocultured ES‐2 cells and HPMCs, stained with phalloidin and DAPI. Scale bars, 10 μm. (*k*) Schematic protocol for coculture invasion assay. (*l*) Length from the bottom of the chamber to the cell layer was evaluated in cocultures of ES‐2 with either untreated or TGF‐β1‐treated HPMCs. (M) Representative 2D and 3D images of cocultured ES‐2 cells and HPMCs. Arrowheads represent an enlarged image of TGF‐β1‐treated HPMCs invading into the collagen gel matrix, followed by ES‐2 cells.

Histological analysis of surgical specimens from patients with peritoneal metastatic dissemination diagnosed with advanced OvCa, revealed fibroblastic cells surrounding tumor cells that were associated with the monolayer of peritoneal MCs. In serial sections, the mesothelial marker, calretinin and the myofibroblast marker, αSMA,^16^ were both positively identified on fibroblastic cells as a component of the tumor microenvironment (Fig. 1 *a* and Supporting Information Figs. S1*a* and S1*b*). These findings suggest that MC‐derived fibroblasts proliferate, and invade the peritoneum, and thus contribute to the tumor microenvironment of OvCa cells during peritoneal dissemination. We defined these cells as OCAMs and evaluated the functional differences that exist between OCAMs and nonactivated MCs.

To investigate morphological changes in MCs within the OvCa tumor microenvironment, we extracted HPMCs that had been cultured with either control growth media or ascites from patients with malignant ovarian tumors. Results show that compared to the cobblestone appearance in the control condition, HPMCs cultured with malignant ascites were spindle‐shaped and presented with increased actin stress fibers in their cytoplasm (Fig. 1 *b*). This mesenchymal change in MCs may have been caused by the release of a soluble growth factor from cancer cells as specific studies have already reported that a variety of factors collected from the conditioned media used to culture OvCa cells induced mesenchymal changes in MCs.^23^ Notably, TGF‐β1 has been recognized as one of the most common mesenchymal inducers of MCs.^16^, ^19^, ^30^ An average of 4.62 ng/ml (range: 0.38–22.34 ng/ml) of TGF‐β1 was detected in the ascites of patients with malignant ovarian tumors (Fig. 1 *c*). TGF‐β1 was also highly expressed in OvCa tissue compared to normal ovarian tissue (Supporting Information Fig. 2*a*) and its level of expression increased as the tumor stage or grade progressed (Supporting Information Figs. S2*b* and S2*c*). In terms of prognosis, overall survival of OvCa patients expressing higher levels of TGF‐β1 was poorer than those with lower expression (Supporting Information Fig. S2*d*). Furthermore, immunoblotting analysis HPMC lysate revealed that αSMA was upregulated after treatment with TGF‐β1, which likely acted *via* the SMAD axis; however, this effect was inhibited by treatment with RI. We confirmed the same effect in HPMCs that had been cultured with malignant ascites; the expression of αSMA and phospho‐SMAD2 were seen to decrease after treatment with RI (Fig. 1 *d*). The expression of genes encoding proteins that are known markers of epithelial–mesenchymal transition (EMT) were also seen to change in HPMCs after treatment with TGF‐β1 (Supporting Information Fig. S1*c*). Further, the cobblestone cellular morphology in HPMCs stimulated by TGF‐β1 or malignant ascites was preserved by treatment with RI (Supporting Information Figs. S1*d* and S1*e*). Additionally, using gelatin zymography, we determined that HPMCs treated with TGF‐β1 exhibited upregulated secretion of MMP 2 and 9 (Fig. 1 *e*). Collectively, these findings suggest that TGF‐β1 is one of the principal inducers of mesenchymal transition and invasive MC phenotypes in peritoneal metastasis of OvCa.

As a functional analysis focusing on the motility and invasiveness of OCAMs, we performed scratch assays and evaluated the proportion of wound confluence with time‐lapse observation over 72 hr (Fig. 1 *f*). Our results indicate that no significant effects on motility were observed after the treatment of HPMCs with TGF‐β1. Alternatively, TGF‐β1‐stimulation enhanced invasiveness in HPMCs compared to cells cultured with control media or media containing TGF‐β1 and RI (Fig. 1 *g*).

To investigate the impact that MC conversion has on the trans‐mesothelial migration of OvCa cells, we developed a coculture system using fluorescently labeled OvCa cells and HPMCs. Fluorescently labeled ES‐2 and SKOV3 cells, stained with CellTracker Green CMFDA dye, were cocultured with HPMCs that had previously been cultured with or without TGF‐β1. The number of OvCa cells attached to the glass after 20 min were then counted (Fig. 1 *h* and Supporting Information Fig. S3*a*). Results show that, compared to cells cocultured with nontreated HPMCs, significantly more OvCa cells in culture with TGF‐β1‐treated HPMCs were seen to migrate into the HPMC monolayer and attached to the glass (Fig. 1 *i* and Supporting Information Fig. S3*b*). Moreover, in three‐dimensional (3D) construction images, OvCa cells exhibited an extended mesenchymal shape in coculture with TGF‐β1‐treated HPMCs compared to the small, rounded shape observed in culture with nontreated HPMCs (Fig. 1 *j* and Supporting Information Fig. S3*c*). While mesenchymal transition of OvCa cells was reported to be caused by several soluble growth factors,^23^ this finding indicates that an adhesive molecule may also greatly influence the mesenchymal characteristics of OvCa cells.

We next established a 3D peritoneal dissemination model to evaluate how OvCa cells invade the extracellular matrix concomitantly with MCs. Untreated and TGF‐β1‐treated HPMCs were plated on collagen gel in trans‐well chambers. Fluorescently labeled ES‐2 cells were cultured on top of the HPMC layer. After culturing with a chemoattractant present in the lower well for 72 hr, we measured the length between the bottom of the chamber and the layer of cells (Fig. 1 *k*). Results revealed that ES‐2 cells cultured with TGF‐β1‐treated HPMCs degraded the collagen gel more strongly than those cultured with untreated HPMCs (Fig. 1 *l*). Moreover, through the examination of a 3D image depicting the cellular invasion patterns, we determined that ES‐2 cells invaded the collagen matrix in a manner similar to that of TGF‐β1‐treated HPMCs (Fig. 1 *m*). Taken together, these findings indicate that OCAMs are capable of influencing the behavior of OvCa cells, as a component of the tumor microenvironment of peritoneal dissemination in OvCa.

### OCAMs induce platinum‐resistance in OvCa cells

**Figure 2:**
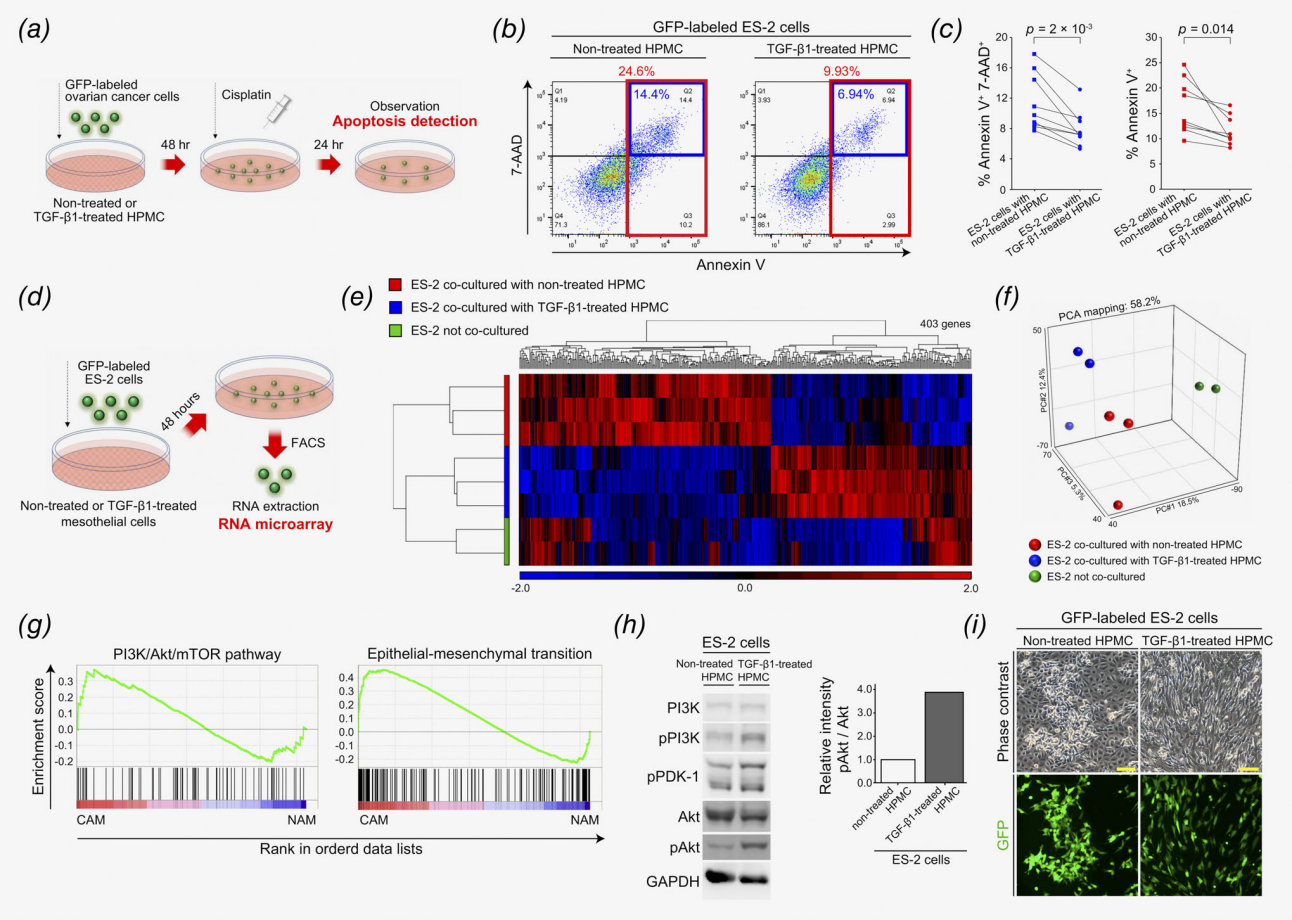
OCAMs induce platinum drug resistance in OvCa cells. (*a*) Schematic representation of the protocol employed to detect the proportion of apoptotic cells in cocultures of OvCa cells and HPMCs. (*b*, *c*) Flow cytometric analysis of Annexin V and 7‐AAD expression in GFP‐labeled ES‐2 cells isolated from coculture with HPMCs. Percentage of both Annexin V and 7‐AAD, or only Annexin V positive cells were presented (*n* = 9). (*d*) Schematic flow diagram depicting the protocol used for RNA microarray analysis. (*e*) A heat map depicting 403 differentially expressed genes in ES‐2 cells cocultured with untreated or TGF‐β1‐treated HPMCs, or in ES‐2 cells alone. (*f*) A principal component analysis (PCA) map illustrating similarities in gene expression between each sample group. (*g*) Gene set enrichment analysis (GSEA) in ES‐2 cells cocultured with TGF‐β1‐treated HPMCs compared to those cultured with untreated HPMCs. This analysis suggests that alterations in genes associated with Akt signaling pathway and the epithelial–mesenchymal transition (EMT) process were identified. (*h*) Immunoblot analysis of ES‐2 cells cocultured with untreated or TGF‐β1‐treated HPMCs, targeting proteins associated with the PI3K/Akt signaling pathway. (*i*) Phase‐contrast images of ES‐2 cells and HPMCs after 48 hr of coculture. Scale bars, 100 μm.

To further evaluate whether cell‐to‐cell crosstalk occurs between OCAMs and OvCa cells, specifically those that persistently survive during peritoneal dissemination, we performed chemo‐sensitivity assays using the coculture system with a platinum agent as a representative first‐line drug for chemotherapy.^31^, ^32^ GFP‐labeled OvCa cells (ES‐2, SKOV3, and OV90) were cocultured with untreated or TGF‐β1‐treated HPMCs. After 48 hr, the cells were treated with cisplatin. After a further 24–48 hr incubation, only the OvCa cells were identified using flow cytometry and apoptotic cells were quantified *via* staining with Annexin V and 7‐AAD (Fig. 2 *a*). The results revealed significantly lower levels of apoptosis in OvCa cells cocultured with TGF‐β1‐treated HPMCs compared to those cultured with untreated HPMCs (Figs. 2 *b* and 2*c* and Supporting Information Fig. S3*d*). These results suggest that OCAMs can induce decreased platinum‐sensitivity in OvCa cells *via* an unknown mechanism involving cell‐to‐cell interactions.

To comprehensively detect differential gene expression between OvCa cells cocultured with untreated HPMCs or TGF‐β1‐treated HPMCs, we isolated only GFP‐labeled ES‐2 cells using FACS after cocultures; total RNA was extracted and RNA microarray analyses were performed (Fig. 2 *d*). Cluster, volcano plots, heat maps, and principal component analysis were performed/generated for the different groups of ES‐2 cells (ES‐2 cells cocultured with untreated or with TGF‐β1‐treated HPMCs, and ES‐2 cells alone without coculture). Similar gene expression patterns were identified within each sample set (Figs. 2 *e* and 2*f* and Supporting Information Fig. S4*a*). Estimated enrichment scores suggest that a large proportion of the identified DEGs encode proteins associated with the PI3K/Akt/mTOR pathway and the EMT process (Fig. 2 *g*). We further confirmed upregulation of phospho‐Akt, phospho‐PI3K and phospho‐PDK‐1 *via* immunoblotting in cultures containing TGF‐β1‐treated HPMCs (Fig. 2 *h*). Moreover, prior to isolating ES‐2 cells, those cocultured with TGF‐β1‐treated HPMCs were spindle‐shaped and relatively evenly dispersed, whereas those cultured with untreated HPMCs exhibited a phenotype similar to epithelial cells and were closely aggregated (Fig. 2 *i*).

### Prognostic relevance of PI3K‐Akt activation and EMT‐related gene signature in OvCa patients

**Figure 3:**
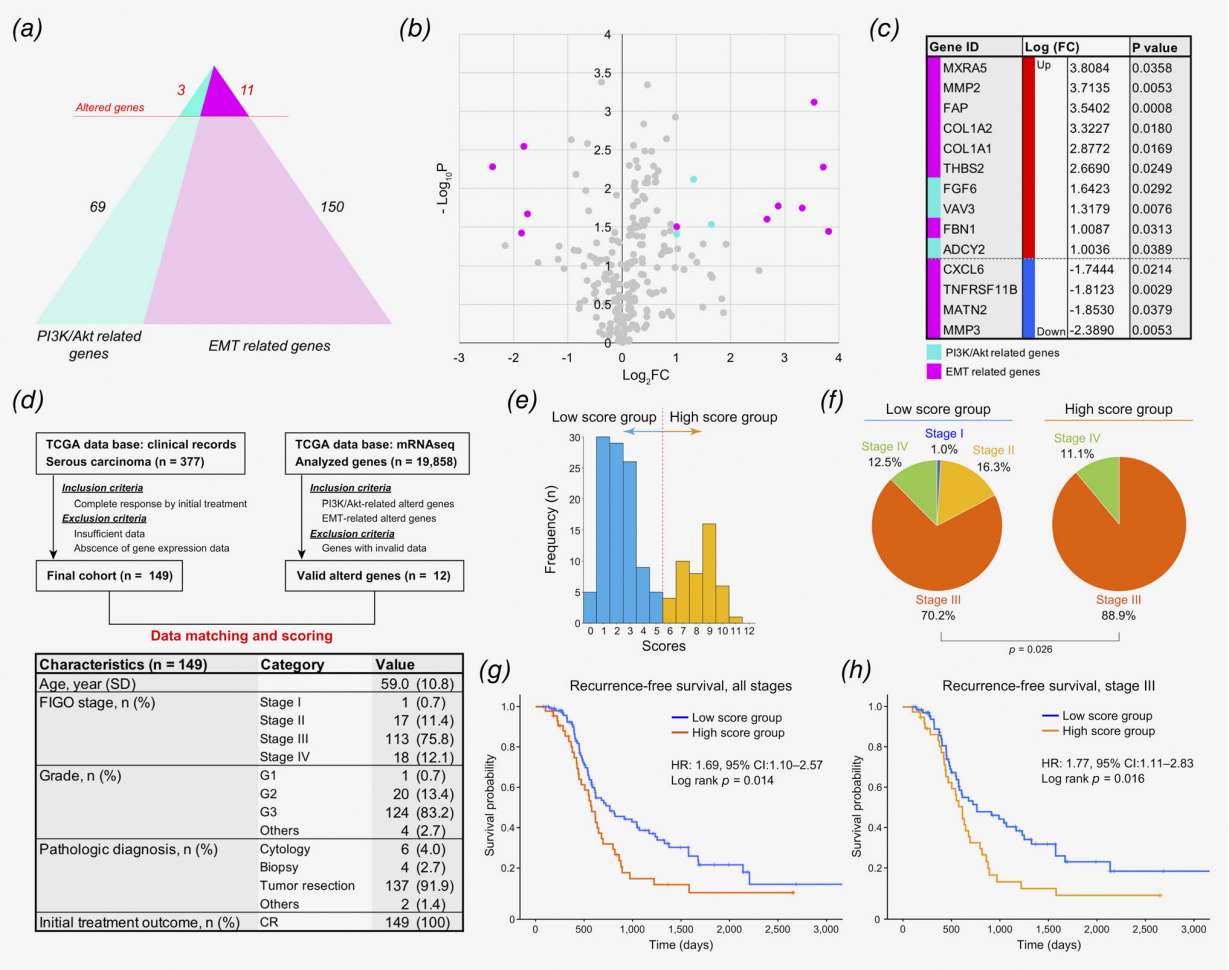
Prognostic relevance of PI3K‐Akt activation and EMT‐related gene signature in ovarian cancer patients. (*a*, *b*) A chart and a volcano plot of the 233 identified genes encoding proteins associated with PI3K/Akt and EMT pathways as determined by GSEA. Fourteen of the genes were significantly differentially expressed in ES‐2 cells cocultured with TGF‐β1‐treated HPMCs compared to those cultured with untreated HPMCs. (*c*) A list of the 14 DEGs indicating the specific fold change and *p*‐value for each gene. (*d*) Flowchart for the scoring of serous carcinoma patients based on transcriptional signature obtained on the TCGA database. The lower panel illustrates baseline characteristics of 149 patients of the selected cohort. (*e*) A representative histogram depicting the scoring for 149 patients. Patients with a minimum of six points were considered to be high scoring, which described approximately one‐third of the patients. (*f*) Pie chart showing the distribution of the clinical staging of the patients within the low and high scoring groups. (*g*, *h*) Progression‐free survival curves for the high and low scoring patient groups depicted for the entire cohort and for Stage III patients, respectively.

The results from the RNA microarray lead us to hypothesize that OvCa cells persistently survive with OCAMs in the peritoneal metastatic tumor microenvironment even after treatment with chemotherapeutic agents including platinum drugs. Consequently, we used a simple mathematical model to assess patient datasets on a public database (TCGA) to predict the clinical impact that OCAMs have on OvCa cells. We identified 14 DEGs with a ≥twofold expression change in genes that encode proteins associated with the PI3K/Akt/mTOR and EMT‐related pathways (3: PI3K/Akt related; 11: EMT‐related; Figs. 3 *a*–3 *c* and Supporting Information Figs. S4*b* and S4*c*). Moreover, from a public clinical dataset (TCGA) that contained the RNA sequences for 377 patients diagnosed with serous carcinomas, we identified 149 patients with OvCa whose tumor completely responded to initial treatment. Using a mathematical model that mimicked the differentially expressed gene pattern as observed in the presence of OCAMs, each patient was assigned a score based on how many of the valid 12 DEGs were identified in their transcriptional signature (Fig. 3 *d* and Supporting Information Fig. S4*d*). A representative histogram of these scores showed that approximately one‐third of the patients were categorized as high scoring (≥6 points) (Fig. 3 *e*). Moreover, we determined that significantly more patients in the high scoring group were reported to have advanced OvCa (Stage III and IV) compared to those in the low scoring group (Fig. 3 *f*). In addition, patients in the high scoring group demonstrated poorer progression‐free survival in all stages as well as in Stage III patients specifically (Figs. 3 *g* and 3*h*). Based on these results, PI3K‐Akt activation and EMT‐related gene signatures, which were similarly induced in ES‐2 cells exposed to OCAMs, were indicative of poor prognosis in OvCa patients.

### FN1 upregulated on the surface of OCAMs induces the activation of Akt signaling in OvCa cells

**Figure 4:**
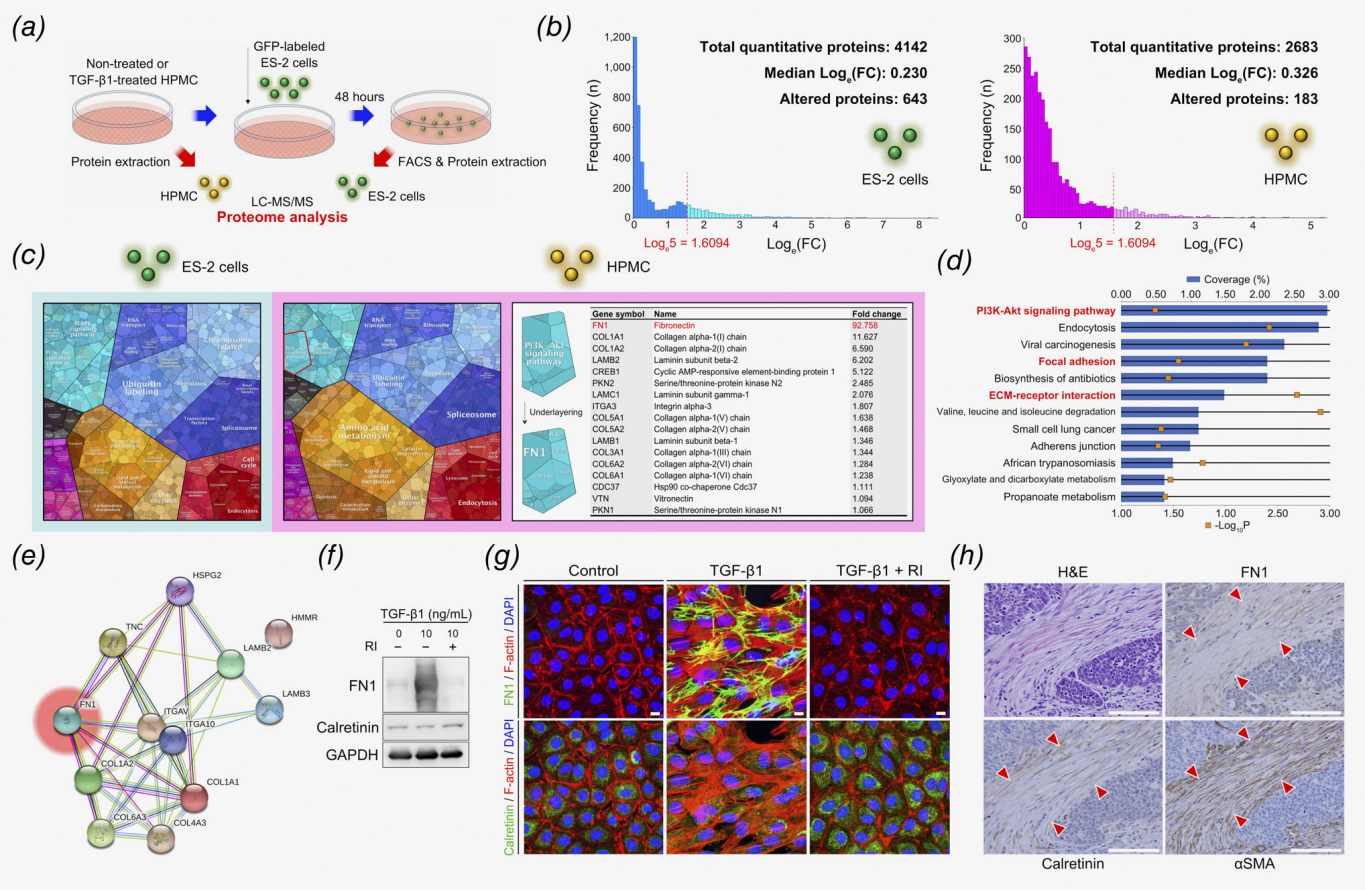
FN1 upregulated on the surface of OCAMs induces the activation of Akt signaling in OvCa cells. (*a*) Schematic depicting the protocol used for protein extraction required for proteomic analysis. (*b*) Histograms depicting the fold changes in identified proteins within ES‐2 cells cocultured with TGF‐β1‐treated HPMCs compared to those cultured with untreated HPMCs, and HPMCs treated with TGF‐β1 compared to those that were untreated. (*c*) Proteomaps depicting the fold changes and associated functions for all identified proteins within ES‐2 cells cocultured with TGF‐β1‐treated HPMCs compared to those cultured with untreated HPMCs, and HPMCs treated with TGF‐β1 compared to those that were untreated. The right panel presents lists and values of fold changes in the expression of proteins identified as being related to the PI3K/Akt signaling pathway in HPMCs treated with TGF‐β1 compared to untreated cells. (*d*, *e*) Pathway enrichment analysis and interactomes created with only proteins that were differentially expressed in both the ES‐2 and HPMCs datasets. (*f*, *g*) Immunoblot analysis and immunofluorescence targeting FN1 and calretinin in HPMCs treated with TGF‐β1 in the presence or absence of RI, Scale bars, 10 μm. (*h*) H&E staining and immunohistochemistry of FN1, calretinin and αSMA in the peritoneal dissemination of OvCa. Arrowheads represent calretinin‐, αSMA‐ and FN1‐positive fibroblastic cells surrounding tumor cells. Scale bars, 100 μm.

Akt signaling, one of the pathways identified as being altered in OvCa cells induced by OCAMs, has been described as being associated with platinum‐resistance in OvCa cells.^33^, ^34^, ^35^, ^36^ To examine if any upstream molecules in the Akt signaling pathway are also affected by OCAMs in OvCa cells, we performed a screening proteomic analysis using liquid chromatography‐mass spectrometry (LC–MS/MS). We first extracted proteins from untreated and TGF‐β1‐treated HPMCs. We then extracted proteins from ES‐2 cells isolated by FACS after coculturing with untreated or TGF‐β1‐treated HPMCs (Fig. 4 *a*). We defined a differentially expressed protein as exhibiting a ≥fivefold change in the geometric mean expression of each sample set. Using these parameters, we detected 643 proteins in ES‐2 cells that were differentially expressed after culturing with TGF‐β1‐treated HPMCs, and 183 differentially expressed proteins were found in HPMCs caused by direct TGF‐β1 stimulation (Fig. 4 *b*). The resulting proteomap images accounted for the entire protein dataset and identified potential protein candidates associated with the altered functions, including those associated with Akt signaling pathway, in both ES‐2 cells and HPMCs. In particular, fibronectin (FN1) was the protein identified as most highly associated with Akt signaling in HPMCs (Fig. 4 *c* and Supporting Information Fig. S5*a*). Moreover, pathway analysis involving the differentially expressed proteins identified in ES‐2 and HPMCs revealed that Akt signaling is significantly affected in ES‐2 cells. Additionally, this analysis highlighted a possible association among focal adhesion, ECM and receptor interactions (Fig. 4 *d*). These results were confirmed using interactome analysis, and FN1 was highlighted as a central effector in these pathways (Fig. 4 *e* and Supporting Information Fig. S5*b*).

Based on our *in silico* results, we were able to identify FN1 within TGF‐β1‐treated HPMCs as a possible inducer of Akt signaling activation in ES‐2 cells. Furthermore, immunoblotting analysis of HPMCs demonstrated that FN1 was highly upregulated by TGF‐β1 (Fig. 4 *f*). These results were confirmed *via* immunofluorescence staining (Fig. 4 *g*). Similarly, histological analysis of OvCa peritoneal metastasis showed FN1 expression in stromal cells, which were also positive for calretinin and αSMA (Fig. 4 *h*). These findings suggest that FN1 found to be upregulated on OCAMs may be one of the principal inducers of Akt signaling activation in OvCa cells *via* direct interaction between these two cell types.

### Induction of Akt signaling in OvCa cells is associated with platinum‐resistance

**Figure 5:**
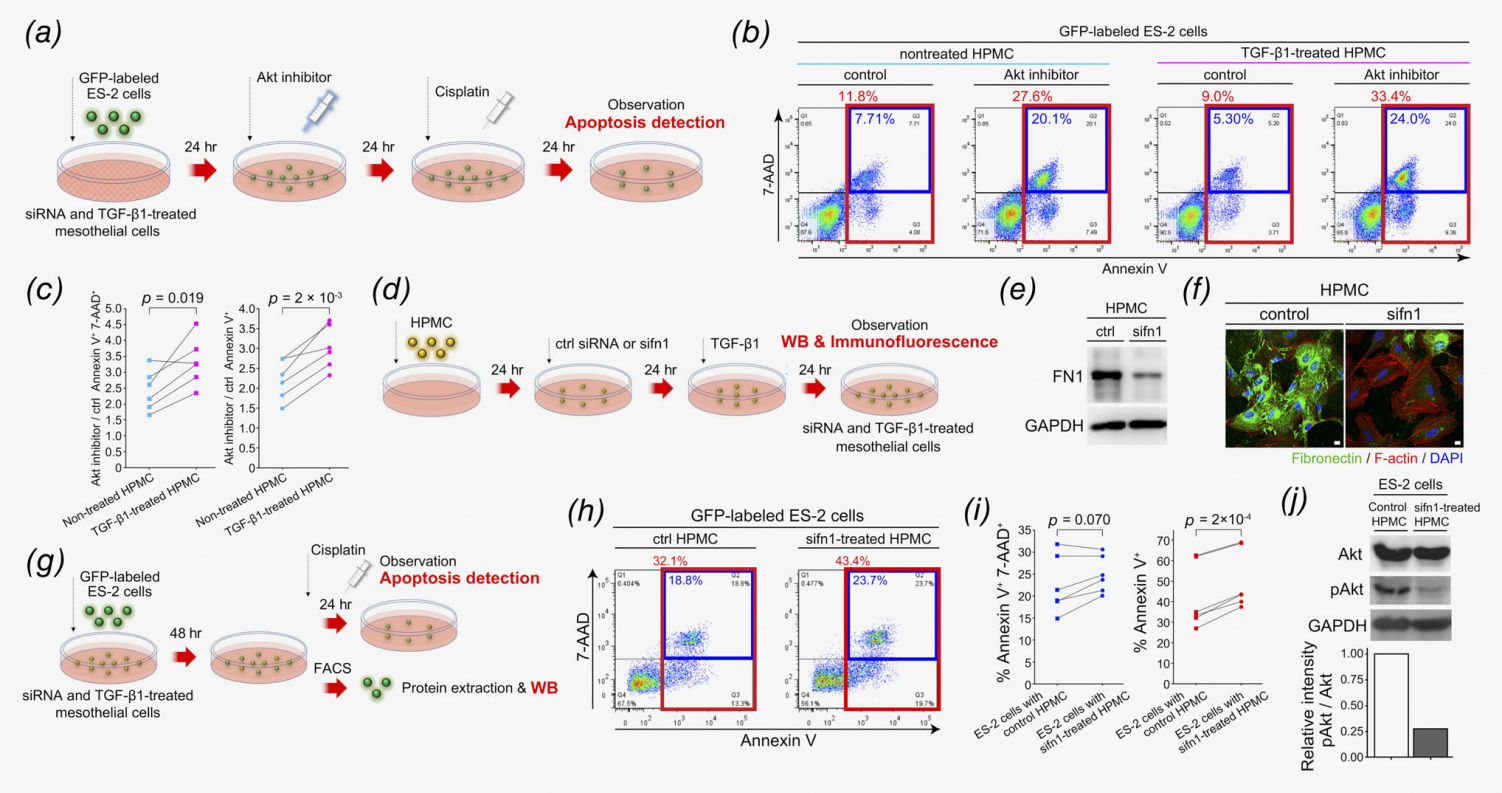
Activation of the FN1‐induced Akt signaling in OvCa cells is associated with platinum drug resistance. (*a*) Schematic image of the protocol used for detection of apoptotic cells after treatment with an Akt inhibitor. (*b*, *c*) Flow cytometric analysis showing the ratio between Akt inhibitor cultures and control cultures in the proportion of Annexin V+ 7‐AAD+ cells or Annexin V+ cells, between cocultures with untreated and TGF‐β1‐treated HPMCs (*n* = 6). (*d*) Schematic depicting the protocol used in treating HPMCs with siRNAs specific for FN1. (*e*, *f*) Immunoblot analysis and immunofluorescence of HPMCs treated with control siRNA or FN1 targeted siRNA. Scale bars, 10 μm. (*g*) Schematic depicting the protocol performed for the detection of apoptotic cells *via* FACS analysis after treatment with FN1‐specific siRNA. (*h*, *i*) Flow cytometric analysis of Annexin V and 7‐AAD expression in GFP‐labeled ES‐2 cells isolated from cocultures with HPMCs treated with control or FN1‐targeted siRNA. The proportion of both Annexin V and 7‐AAD, or Annexin V positive cells are illustrated (*n* = 6). (*j*) Immunoblot analysis of ES‐2 cells cocultured with HPMCs treated with control or FN1‐targeted siRNA.

Our proteomic analysis suggests that upregulation of FN1 on OCAMs may induce platinum‐resistance *via* activating Akt signaling in OvCa cells. To confirm the association between platinum‐resistance and Akt signaling, we firstly evaluated the proportion of apoptotic ES‐2 cells in cocultures with untreated and TGF‐β1‐treated HPMCs both in the presence and absence of an Akt inhibitor. Briefly, GFP‐labeled ES‐2 cells were plated on untreated, and TGF‐β1‐treated HPMCs, and cocultured for 24 hr. We then added Akt inhibitor and cultured for an additional 24 hr. Cisplatin was then added to the reaction wells and cultured for 24 hr. Finally, ES‐2 cells were isolated *via* flowcytometry and apoptotic cells were detected using Annexin V and 7‐AAD staining (Fig. 5 *a*). When examining the proportion of cells that stained positive for both Annexin V and 7‐AAD, we observed more apoptotic cells in cultures containing ES‐2 cells with an Akt inhibitor compared to those without the inhibitor. Similar results when observed in cells that stained positive for only Annexin V (Supporting Information Fig. S6*a*). Further, when examining the role of Akt signaling in inducing platinum resistance in ES‐2 cells, we compared the ratio of cells in culture with Akt inhibitor compared to controls, that stained positive for both Annexin V and 7‐AAD to those that were only Annexin V+ in cocultures of untreated and TGF‐β1‐treated HPMCs. The results revealed that significantly higher ratios of Akt inhibitor: controls were observed in cultures with ES‐2 cells and TGF‐β1‐treated HPMCs compared to the paired control samples (Figs. 5 *b* and 5*c*). These results suggest that activation of Akt signaling induced by TGF‐β1‐treated HPMCs may be involved in decreased platinum‐sensitivity in ES‐2 cells.

We next investigated whether the upregulation of FN1 specifically on OCAMs influenced platinum‐resistance and activation of the Akt signaling pathway in OvCa cells. To validate an experimental model using siRNA knockdown, HPMCs were transfected with control siRNA or siRNA specific for FN1 (siFN1) and stimulated with TGF‐β1 (Fig. 5 *d*). We confirmed the downregulation of FN1 expression in TGF‐β1‐stimulated HPMCs treated with siFN1 *via* immunoblotting and immunofluorescence (Figs. 5 *e* and 5*f*, and Supporting Information Figs. S6*b* and S6*c*). Additionally, GFP‐labeled ES‐2 cells were plated on siRNA‐ and TGF‐β1‐treated HPMCs and cultured for 48 hr, followed by treatment with cisplatin for 24 hr. ES‐2 cells were then isolated *via* flow cytometry and the proportion of apoptotic cells was quantified using Annexin V and 7‐AAD staining (Fig. 5 *g*). The results showed a significant increase in apoptotic ES‐2 cells cocultured with siFN1‐treated HPMCs compared to those cultured with control siRNA‐treated HPMCs (Figs. 5 *h* and 5*i* and Supporting Information Fig. S6*d*). We also confirmed a decrease in the expression level of phospho‐Akt in ES‐2 cells cocultured with siFN1‐treated HPMCs prior to the addition of cisplatin (Fig. 5 *j* and Supporting Information Fig. S6*e*). Collectively, these results suggest that FN1 on OCAMs induces decreased platinum‐sensitivity *via* Akt signaling in OvCa cells.

### Akt signaling is activated in OvCa cells *via* TGF‐β1 stimulation in the peritoneum of mice

**Figure 6:**
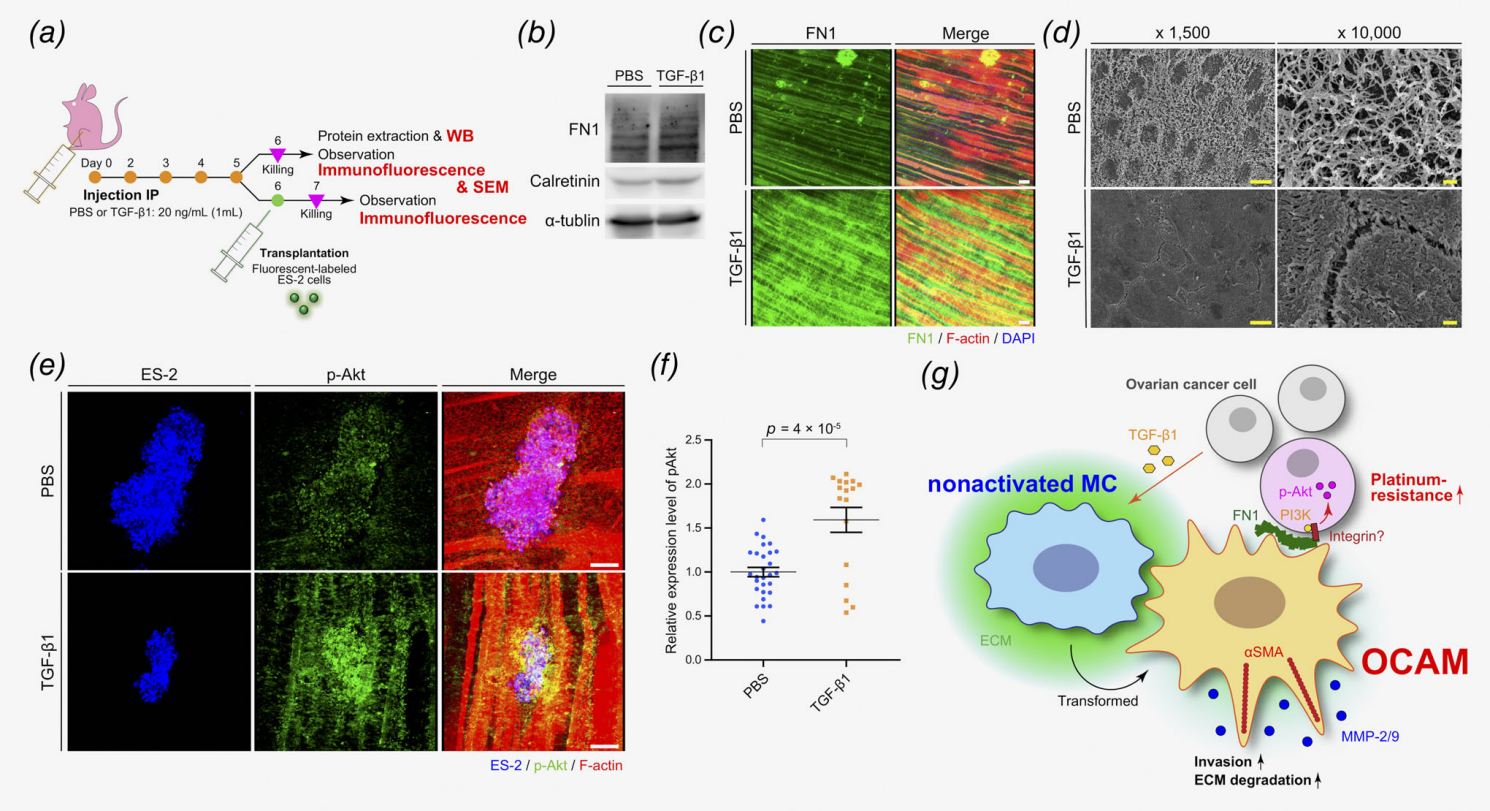
Akt signaling is activated in OvCa cells *via* TGF‐β1 stimulation in the peritoneum of mice. (*a*) Schematic depicting of the protocol used for *in vivo* mouse experiments to confirm the proposed mechanism of platinum drug resistance. (*b*, *c*) Immunoblot analysis and immunofluorescence of the mice peritoneal surface treated with control PBS or TGF‐β1. (*d*) Representative images from SEM of the peritoneal surface treated with control PBS or TGF‐β1. Scale bars, 100 μm. (*e*) Representative images of immunofluorescence showing xenograft tumors in the peritoneum of mice treated with control PBS or TGF‐β1. Scale bars, 100 μm. (*f*) Quantitative analysis of the phospho‐Akt expression in clusters of xenograft tumor cells in mice injected with control PBS (*n* = 28) compared to those injected with TGF‐β1 (*n* = 17). (*g*) A hypothetical model describing the cell‐to‐cell crosstalk between OvCa cells and OCAMs in the tumor microenvironment during peritoneal dissemination.

We developed an *in vivo* experimental mouse model of peritoneal dissemination to evaluate the proposed mechanism for platinum drug resistance, as described above. First, to evaluate whether TGF‐β1 upregulates FN1 on the surface of the peritoneum in mice, PBS or TGF‐β1 were intraperitoneally injected into the abdominal cavity of mice once a day for 5 days. We evaluated the morphological differences that developed in the peritoneum as well as FN1 expression levels (Fig. 6 *a*). Immunoblotting of the protein extracted from the peritoneal surface revealed higher FN1 expression in mice treated with TGF‐β1 compared to those injected with PBS (Fig. 6 *b*). These results were confirmed using immunofluorescent staining (Fig. 6 *c*). Scanning electron microscopy was performed to detect morphological changes in the peritoneum. We observed thickly growing microvilli on the peritoneal surface of control mice, while rough and short microvilli were confirmed on the surface of the peritoneum in TGF‐β1‐stimulated mice. We also detected collapsed cell‐to‐cell junctions in the peritoneum of TGF‐β1‐stimulated mice (Fig. 6 *d* and Supporting Information Fig. S7A). These observations suggest that TGF‐β1 may induce morphological changes and FN1 expression on the peritoneal surface.

Next, we intraperitoneally injected fluorescently labeled ES‐2 cells into mice after the stimulation with either PBS or TGF‐β1 (Fig. 6 *a*). Using this experimental model, we were able to directly observe metastatic tumor cells on the surface of the peritoneum using stereoscopic microscopy (Supporting Information Fig. S7*b*). We resected mice peritoneum 24 hr after injection and fixed the samples. Spotted lesions on xenograft tumors were confirmed with a stereoscopic microscope and were observed *via* immunofluorescence that targeted the expression of phospho‐Akt. Compared to PBS‐treated control mice, the expression of phospho‐Akt was significantly upregulated in tumor cells on the peritoneum of TGF‐β1‐treated mice (Figs. 6 *e* and 6*f*). Taken together, the results from our study suggest that OCAMs are present within the tumor microenvironment of peritoneal metastasis in OvCa, and express FN1 causing platinum‐resistance in OvCa cells *via* the activation of the Akt signaling pathway (Fig. 6 *h*).

## Discussion

Most patients with OvCa are diagnosed at an advanced stage, and present with peritoneal metastasis.^3^, ^6^ Response to the initial treatment is generally favorable with approximately 50% of patients experiencing remission.^37^, ^38^ However, more than half of those that enter remission will later develop tumor recurrence,^39^, ^40^, ^41^ suggesting the presence of persistent occult tumor metastasis in the peritoneum, which is challenging to eradicate, even after administration of conventional platinum‐based chemotherapy.^42^ These clinical and pathophysiological characteristics of OvCa suggest that the peritoneum acts as an anchoring point for metastatic tumor cells, which promotes and maintains the robust survival of OvCa cells. It is, therefore, important to identify novel therapeutic targets against peritoneal metastasis and its tumor microenvironment in advanced OvCa.

CAFs, potentially originating from various types of cells, appear in all stages of cancer and have pivotal roles in creating tumor microenvironments.^25^ As it relates to OvCa, a variety of CAFs have been reported to comprise the tumor stroma, thereby contributing to the development of stromal heterogeneity.^43^, ^44^ Moreover, it has been recognized that mesenchymal conversion of MCs is associated with tissue fibrosis in peritoneal dialysis.^45^, ^46^ It has also been proposed that the transition of mesothelial cells, predominantly initiated by TGF‐β1, has an important role in the development of peritoneal metastasis.^16^, ^17^, ^19^

In the current study, histological analysis revealed the presence of myofibroblasts, consistently associated with peritoneal MCs. This finding suggests that OCAMs proliferate and, together with OvCa cells, invade the peritoneal tissues. Moreover, OCAMs and OvCa cells were seen to effectively penetrate the ECM of the peritoneum, which is supported by a previous report.^47^ Since OCAMs and OvCa cells coexist in the tumor microenvironment, it is reasonable to postulate that they are mutually associated *via* direct cell‐to‐cell crosstalk. We, therefore, examined the mechanism by which OCAMs influence persistent survival of OvCa cells, thereby causing further promotion of peritoneal metastasis and the development of platinum anticancer drug resistance.

The primary finding in our study suggests that OvCa cells acquire platinum‐resistance *via* activation of Akt signaling, which is induced by FN1 on the surface of OCAMs. Pathway analysis identified Akt signaling in OvCa cells as significantly altered after stimulation by OCAMs. We also determined that patients with tumors that had a similar gene expression pattern to OvCa cells cocultured with OCAMs are at greater risk of their cancer recurring after the initial tumor remission. Although we focused primarily on the Akt signaling pathway, EMT‐related gene signatures were also found to be important with the cell‐to‐cell crosstalk. Since EMT also plays a significant role in cisplatin resistance in OvCa,^48^ the decreased sensitivity of OvCa cells in our *in vitro* coculture experiments may also be caused by the altered EMT‐related gene signatures. In addition, the 14 genes used in the model primarily belong to the Tothill's C5 subtype which is associated with poor prognosis.^49^ Survival outcome results between the two groups may also stem from these OvCa pathological backgrounds. Although these results were based on simple mathematical modeling, it nonetheless provides a novel translational method for the analysis of multiple variables related to gene expression and pathway analysis.

Furthermore, using LC–MS/MS, we identified interactions *via* FN1 on the surface of OCAMs, as an upstream effector of Akt signaling in OvCa cells. Although our proteomic analysis was designed in such a way as to screen possible candidates associated with our target interactions, which may have added a certain level of bias to the study, we confirmed the role of FN1 in the development of OvCa cell platinum‐resistance with siRNA experiments. And thus, we are confident in our findings that FN1 contributes to platinum‐resistance by directly activating the Akt signaling pathway. Taken together, these findings clearly suggest that OCAMs are a critical component in the tumor microenvironment that promotes the progression of peritoneal metastasis of OvCa.

Platinum agents are the key drugs used in chemotherapeutic treatment of OvCa,^31^, ^32^ of which effectiveness is estimated *via* a treatment‐free interval that can clearly predict the prognosis of patients with advanced disease.^11^, ^12^ It is also known that Akt signaling pathway is associated with platinum resistance in OvCa cells.^33^, ^34^, ^35^, ^36^ Moreover, FN1 has been described as having a pivotal role in the progression of OvCa cells,^50^, ^51^ and is also reported to induce resistance for specific chemotherapeutic agents.^52^, ^53^ Although few reports have described a direct association between platinum‐resistance and FN1 expression in stromal cells of peritoneal metastasis, signal transduction from FN1 to Akt *via* integrin and PI3K has been recognized as a critical pathway in cancer progression.^54^, ^55^ Adding to this body of research, we demonstrated that activation of the Akt signaling pathway, induced by FN1 interactions, was associated with platinum‐resistance in OvCa cells in direct contact with OCAMs. Thus, if we can inhibit mesothelial conversion to OCAMs or inhibit the FN1/Akt signaling pathway axis, we may be able to effectively control the development of peritoneal metastatic tumors even with the use of conventional platinum‐based chemotherapy.

Possible limitations of this present study include other major or minor confounding factors, which may have also been responsible for stimulation of OCAMs or OvCa cells, and may have functioned to influence the phenotype of the cells throughout the study. To improve the robustness of the results, we primarily employed HPMCs from different patients and repeated these experiments multiple times. However, further studies are required to elucidate the detailed mechanism and significance of OCAMs role in peritoneal metastasis of OvCa cells.

## Conflict of interest

None declared.

## Supporting information


**Appendix S1**: Supporting information
