# Supplementary material for: Ovarian cancer‐associated mesothelial cells induce acquired platinum‐resistance in peritoneal metastasis via the FN1/Akt signaling pathway
Source: Int J Cancer. 2020 Jan 24;146(8):2268–80. doi: 10.1002/ijc.32854 (PMC7065188; doi:10.1002/ijc.32854)
Supplement: Supplementary file 1 — Appendix S1: Supporting information [file IJC-146-2268-s001.pdf]

Ovarian cancer-associated mesothelial cells induce acquired platinum-resistance in peritoneal metastasis via the FN1/Akt signaling pathway

Masato Yoshihara, Hroaki Kajiyama, Akira Yokoi, Mai Sugiyama, Yoshihiro Koya, Yoshihiko Yamakita, Wenting Liu, Kae Nakamura, Yoshinori Moriyama, Hiroaki Yasui, Shiro Suzuki, Yusuke Yamamoto, Carmela Ricciardelli, Akihiro Nawa, Kiyosumi Shibata, and Fumitaka Kikkawa

Contents

- Supplementary Materials & Methods
- Supplementary tables
- Supplementary figures
- Supplementary references

SUPPLEMENTARY MATERIALS & METHODS

Immunohistochemistry analysis

Histological samples were collected from patients with OvCa in accordance to the guidelines established by the Ethics Committee of Nagoya University. Methods for sample preparation were described previously [1]. Sections from paraffin-embedded tissue blocks (4-µm thick) were stained with anti-Calretinin (ab702, 1:100, Abcam), anti-αSMA (ab7817, 1:100, Abcam), and anti-FN1 (ab194395, 1:100, Abcam) antibodies. A negative control, consisting of blocking buffer without a primary antibody, was included on all sections.

Immunofluorescence

Samples were fixed with 4% formaldehyde in phosphate-buffered saline (PBS), permeabilized with 0.1% Triton X-100 in PBS and stained with 4',6-diamidino-2-phenylindole (DAPI), phalloidin (ab176759, Abcam) with or without each antibody, namely, FN1 (ab194395, Abcam), calretinin (ab133316, Abcam), and phosphor-Akt (ab81283, Abcam). The cells were observed under a laser confocal microscope.

ELISA assay for quantification of TGF-β1

The expression of TGF-β1 was quantified in the ascites from patients with malignant ovarian tumors using ELISA kits (R&D Systems) according to the manufacturer's protocols. Experiments were performed in duplicate.

Gelatin zymography

HPMCs were seeded into 6-well collagen-coated plates and incubated until 100% confluence was reached. After treatment with TGF-β1, with or without RI, for 72 hours, cells were washed and cultured with fresh RPMI media supplemented with 1% fetal bovine serum (FBS) for 72 hours. Supernatants were collected and centrifuged for zymography, which was performed as described previously [2].

Immunoblot analysis

Sodium dodecyl sulfate-polyacrylamide gel electrophoresis (SDS-PAGE) and western blotting were performed as previously described [3]. The primary antibodies were used at the following dilutions: αSMA (ab7817, Abcam, 1:200), SMAD2 (#5339, CST, 1:1,000), phospho-SMAD2 (#3108, CST, 1:1,000), GAPDH (#2118, CST, 1:1,000), PI3K (#3011, CST, 1:1,000) phospho-PI3K (#4228, CST, 1:1,000), phospho-PDK1 (#3438, CST, 1:1,000), pan-Akt (#4691, CST,

1:1,000), phospho-Akt (#4060, CST, 1:1,000), FN1 (sc-18825, Santa Cruz Biotechnology, 1:200), and calretinin (ab133316, Abcam, 1:1,000). The immunoreactive signals were detected via enhanced chemiluminescence (ECL) (#RPN2236) using the ImageQuant LAS 4000 mini kit (GE Healthcare, Buckinghamshire, UK).

Quantitative real-time PCR

Total RNA extraction and sample preparation were principally performed as previously reported [3]. Complementary DNA (cDNA) was synthesized from 1 μg of total RNA using PrimeScript™ II 1st strand cDNA Synthesis Kit with a random primer. Expression levels were normalized to GAPDH expression. Sequences of the primers are listed separately (Supplementary Table 1).

Time lapse scratch assay

HPMCs were plated on collagen-coated 96-well ImageLock plates (Essen BioScience) and cultured until 100% confluence was attained. After treatment with TGF-β1, with or without RI, for 72 hours, a scratch was made in each well using a WoundMaker (Essen BioScience). For the invasion assays, cells were covered with Matrigel (354320, Corning) after creating the

scratch. Progress in the closure process was observed every one hour using the IncuCyte Live-Cell Imaging System.

Trans-mesothelial migration and co-culture invasion assays

To examine the trans-mesothelial migration patterns, HPMCs were plated on collagen-coated glass coverslips and cultured until confluence was achieved. The cells were then cultured with or without 10 ng/mL of TGF-β1 for 72 hours. CMFDA (CellTracker Green, Thermo Fisher Scientific)-stained ES-2 or SKOV3 cells were plated on the HPMC layer in serum-free RPMI media. After 20 minutes of co-culture, unbound cells were washed away, and photographs were taken. The number of CMFDA-stained ES-2 or SKOV3 cells attached to the glass were quantified. Next, the 3D invasion assay was performed using a trans-well chamber (#3422 Corining). HPMCs were plated on pre-coated collagen gel (3 mg/mL) and were cultured, with or without, TGF-β1 for 72 hours in the presence of an MMP inhibitor (#142880-36-2, Cayman Chemical). HPMCs were then stained with CMTPX (CellTracker Red, Thermo Fisher Scientific), and CMFDA-stained ES-2 cells were plated and incubated with serum-free RPMI media in the inserted-well, and with media containing 10% FBS and 50 ng/mL of EGF, as a chemoattractant, in the lower well for 72 hours. Samples were then fixed and photographed

with a laser confocal microscope, allowing for the construction of 3D images using Imaris software.

Public database analysis

The association between TGF-β1 mRNA expression and clinical features of tissues and ovarian cancer patients were analyzed using a microarray gene expression database of ovarian cancer subtypes, CSIOVDB [4].

RNA microarray analysis

Total RNA was amplified and labelled with Cy3 using a one-color Low Input Quick Amp Labeling Kit (Agilent Technologies), according to manufacturer's instructions. Briefly, 100 ng of total RNA was reverse-transcribed to double-stranded cDNA using a poly dT-T7 promoter primer. Primer, template RNA, and quality-control transcripts of known concentrations and qualities were first denatured at 65 °C for 10 minutes and incubated for 2 hours at 40 °C with 5× first-strand buffer, 0.1 M dithiothreitol, 10 mM deoxynucleotide triphosphate mix and AffinityScript RNase Block Mix. The AffinityScript enzyme was inactivated at 70 °C for 15 minutes. cDNA products were then used as templates for *in vitro* transcription to generate

fluorescent complementary RNA (cRNA). cRNA products were mixed with a transcription master mix in the presence of T7 RNA polymerase and Cy3-labeled CTP (cytidine 5′-triphosphate) and incubated at 40 °C for 2 hours. Labelled cRNA was purified using RNeasy Mini Spin Columns (Qiagen) and eluted in 30 µL of nuclease-free water. After amplification and labelling, cRNA quantity and cyanine incorporation were determined using a NanoDrop ND-1000 spectrophotometer and an Agilent Bioanalyzer, respectively. For each hybridization, 0.60 µg of Cy3-labelled cRNA was fragmented and hybridized at 65 °C for 17 hours to an Agilent SurePrint G3 Human GE v3 8x60K Microarray (design ID: 072363). After washing, the microarray chips were scanned using an Agilent SureScan microarray scanner. Intensity values of each scanned feature were quantified using Agilent Feature Extraction software version 11.5.1.1, which performs background subtractions. We only used features that were flagged with no errors (detected flags) and excluded features that were not positive, not significant, not uniform, not above background, saturated, or population outliers (compromised and no detected flags). Normalization was performed using Agilent GeneSpring version 14.8 (per chip: normalization to 75_{th} percentile shift). There is a total of 58,201 probes on the Agilent SurePrint G3 Human GE v3 8x60K Microarray (design ID: 072363) without control probes. The altered transcripts were quantified using the comparative method. The intensity values

were log2-transformed and imported into the Partek Genomics Suite 6.6 (Partek Inc., Chesterfield, MO, USA).

siRNA Transfection

Cells were transfected with 10 nmol/L of control siRNA (#4390843, Ambion) or with siRNA targeting fibronectin (FN1) (s5321, s5323 (#2), Ambion) and 0.5% HiPerFect (Qiagen) in accordance with the manufacture's protocols. Detailed sequences of the siRNAs used for the experiments are listed in Supplementary Table S1.

Scanning electron microscopy

Samples were fixed in 2% glutaraldehyde in 0.1 M phosphate buffer (pH 7.4). Tissues were treated with 2% osmium tetroxide (OsO4) in the same phosphate buffer. Fixed specimens were washed and dehydrated in a graded series of ethanol, replace in tri-butyl alcohol. Samples were pre-impregnated and freeze-dried with tert-butyl alcohol (VFD-21S Shinkuu device Ltd, Ibaraki, Japan). Samples were coated with Osmium Plasma Coater (NL-OPC80NC Filgen Ltd, Aichi, Japan). Sections were analyzed using a Jeol JSM-7610F Scanning electron microscope.

Table S1. Sequence of primers and siRNA used in the study.

qRT-PCR	Forward 5'-3'	Reverse 5'-3'
E-cadherin	TGAGTGTCCCCCGGTATCTTC	CAGTATCAGCCGCTTTCAGATTT
N-cadherin	TGCTGTTTTGGACCGAGAATCACC	CAGCGTTCCTGTTCCACTCATAGG
Snail	CCTCAAGATGCACATCCGAAG	ACATGGCCTTGTAGCAGCCA
Slug	ATGAGGAATCTGGCTGCTGT	CAGGAGAAAATGCCTTTGGA
Twist1	CACCCAGTCGCTGAACGAGGC	CTGCAGCTTGCCATCTTGGAGTC
Twist2	GGCGCAAGTGGAATTGGGATG	CCCTTCTCTCGACGCTGGTG
Zeb1	GCACAACCAAGTGCAGAAGA	CATTTGCAGATTGAGGCTA
Zeb2	CCAGCGGAAACAAGGATTTCAG	ACAGGAGTCGGAGTCTGTCA
VEGF	CTCACCGCCTCGGCTTGTCACA	CCTGGTGGACATCTTCCAGGAGTA
MMP2	GTGGATGCCGCCTTTAACTGGAG	GATGAGCTTGGGGAAGCCAGG
MMP9	GACGTGAAGGCGCAGATGGTG	GGAACTCACGCGCCAGTAGAAG

siRNA	5'-3'
siFN1	GCCCGGUUGUUAUGACAAUtt
siFN1#2	GGUUUUAACUGCGAGAGUAtt

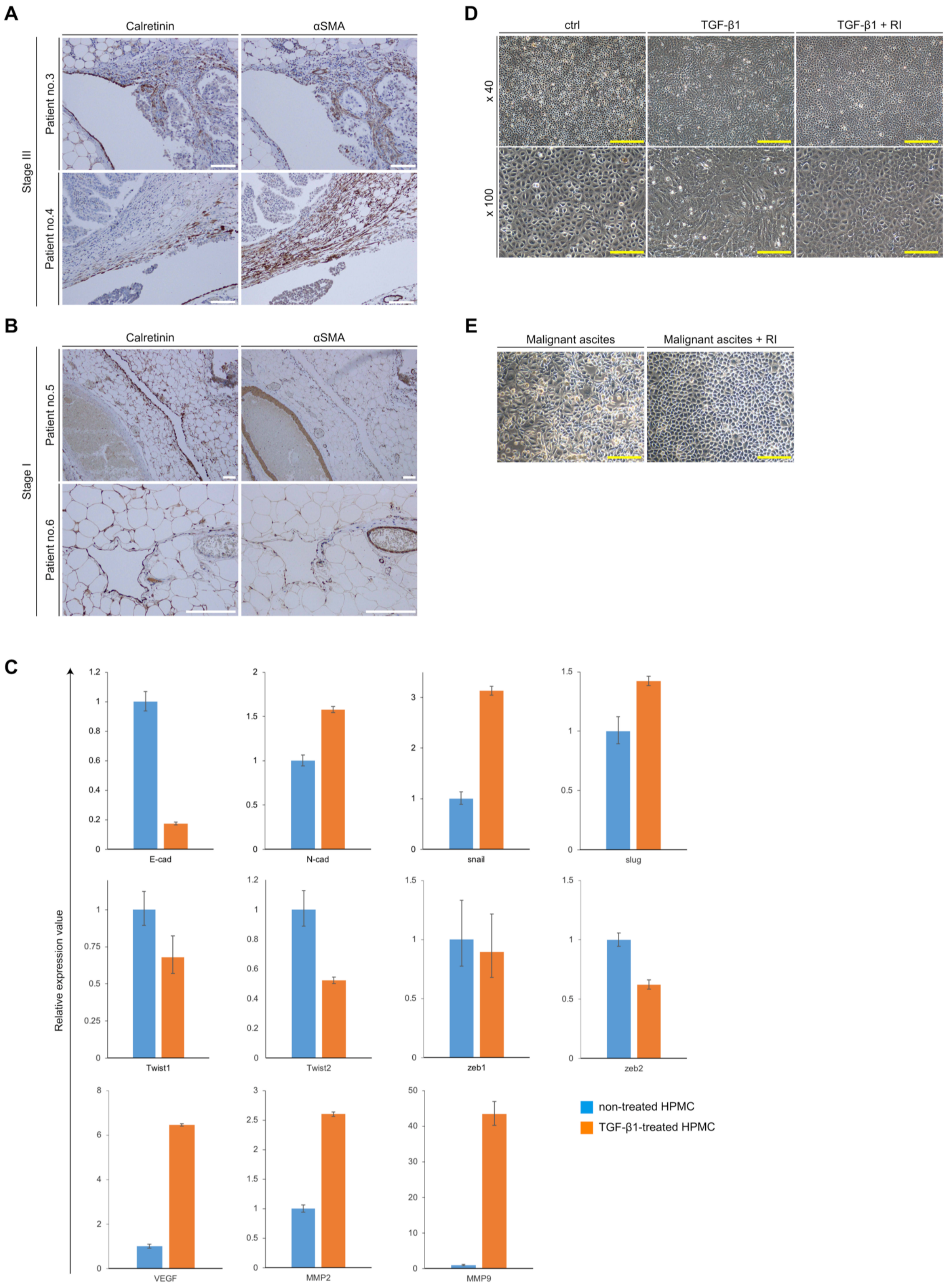

Figure S1. OCAMs characteristics

A) Representative images of immunohistochemistry staining for calretinin and αSMA in peritoneal dissemination of OvCa in Patient 3 and Patient 4. Scale bars, 100 μm. B) Representative images of immunohistochemistry staining for calretinin and αSMA in the omentum of stage I OvCa in Patient 5 and Patient 6. C) Results of qRT-PCR in HPMCs cultured with or without TGF-β1, expression of EMT-related genes were specifically quantified. D) Phase contrast images of non-treated or TGF-β1-treated HPMCs cultured with or without a TGF-β1 receptor inhibitor (RI). Scale bars, 500 μm (Upper 3 images), 200 μm (lower 3 images). E) Phase contrast images of HPMCs treated with ovarian cancer malignant ascites and cultured with or without RI. Scale bars, 200 μm.

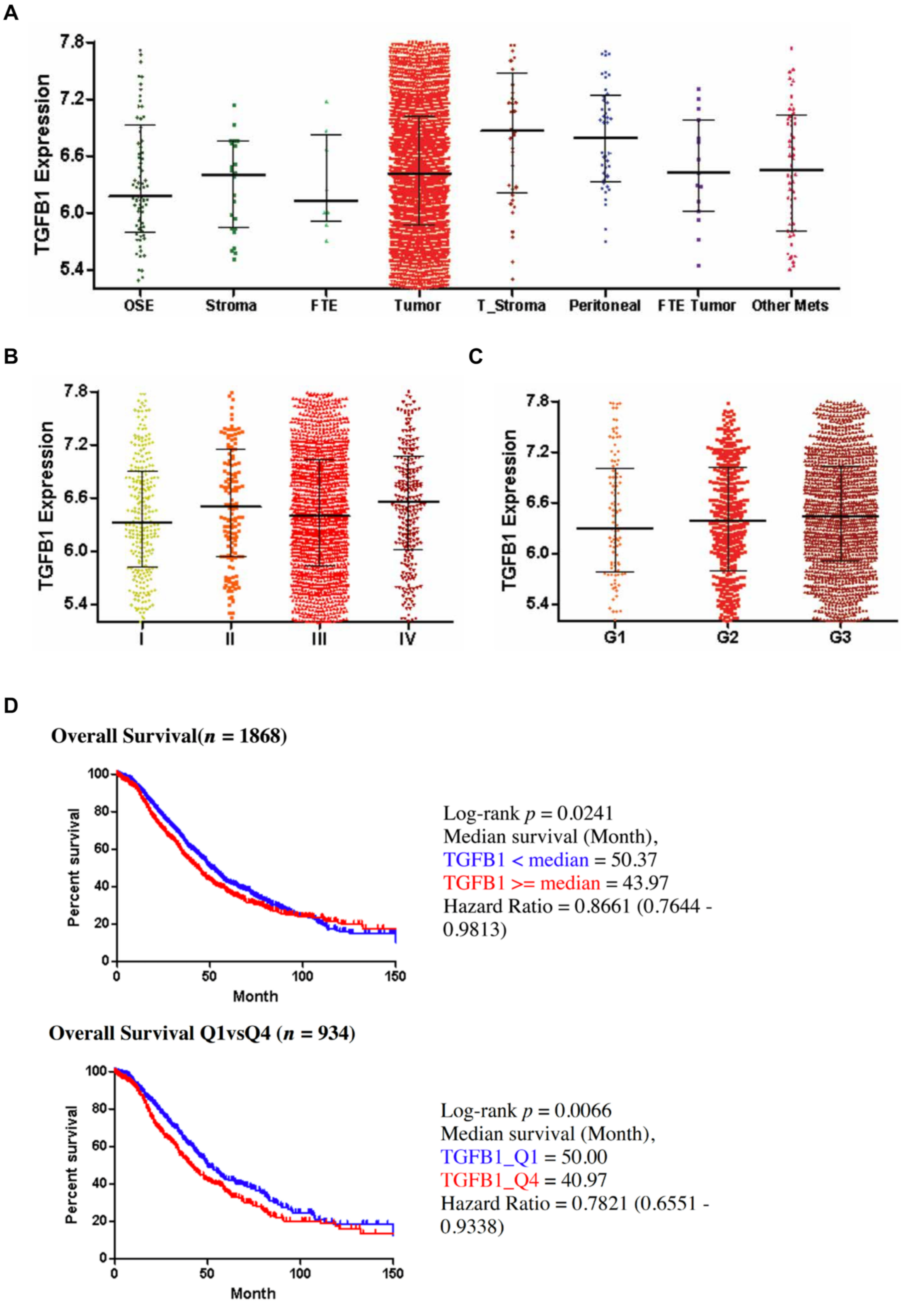

Figure S2. High expression of TGF-β1 is associated with poor prognosis of OvCa
A) TGF-β1 mRNA expression levels in OvCa and normal tissues. B, C) TGF-β1 mRNA expression levels according to stage or grade. D) Overall survival of the patients with OvCa according to TGF-β1 mRNA expression levels.

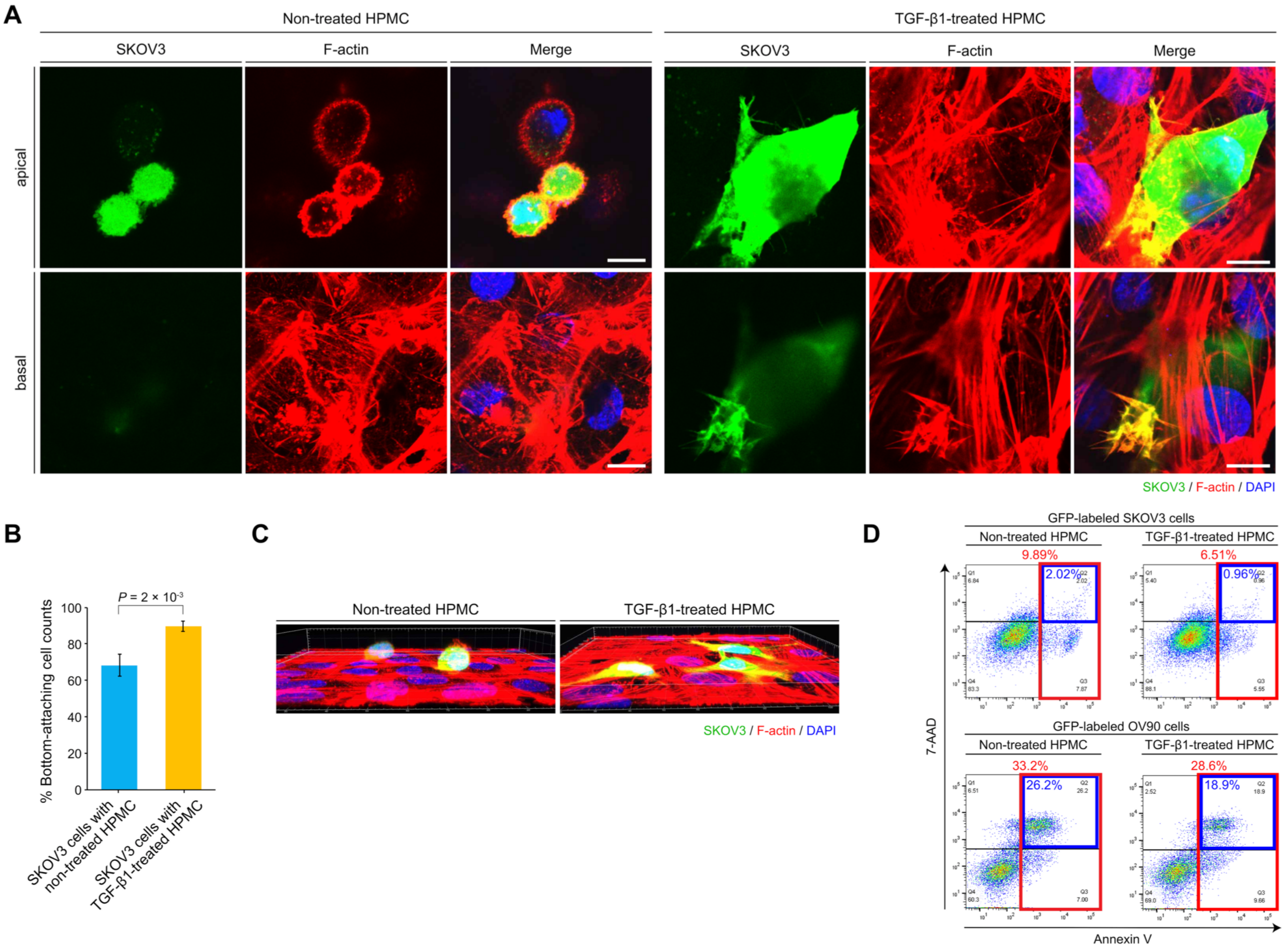

Figure S3. Co-culturing of SKOV3 and OV90 cells and HPMCs

A) Representative image of immunofluorescence in a trans-mesothelial migration assay, stained with phalloidin and DAPI. Apical images were taken of the top layer of HPMCs, while basal images were of the bottom layer. Glass-attaching cells were defined as those located at both apical and basal areas of the images. Scale bars, 100 μm. B) Percentage of SKOV3 cells that were adhered to the bottom of the slide following co-culturing with HPMCs either treated or untreated with TGF-β1. C) Representative 3D depiction of SKOV3 cells adhered to the bottom of the slide in co-culture with HPMCs treated with control media or with media containing TGF-β1, stained with phalloidin and DAPI. D) Flow cytometric analysis of Annexin V and 7-AAD expression in GFP-labeled SKOV3 and OV90 cells isolated from co-culture with HPMCs.

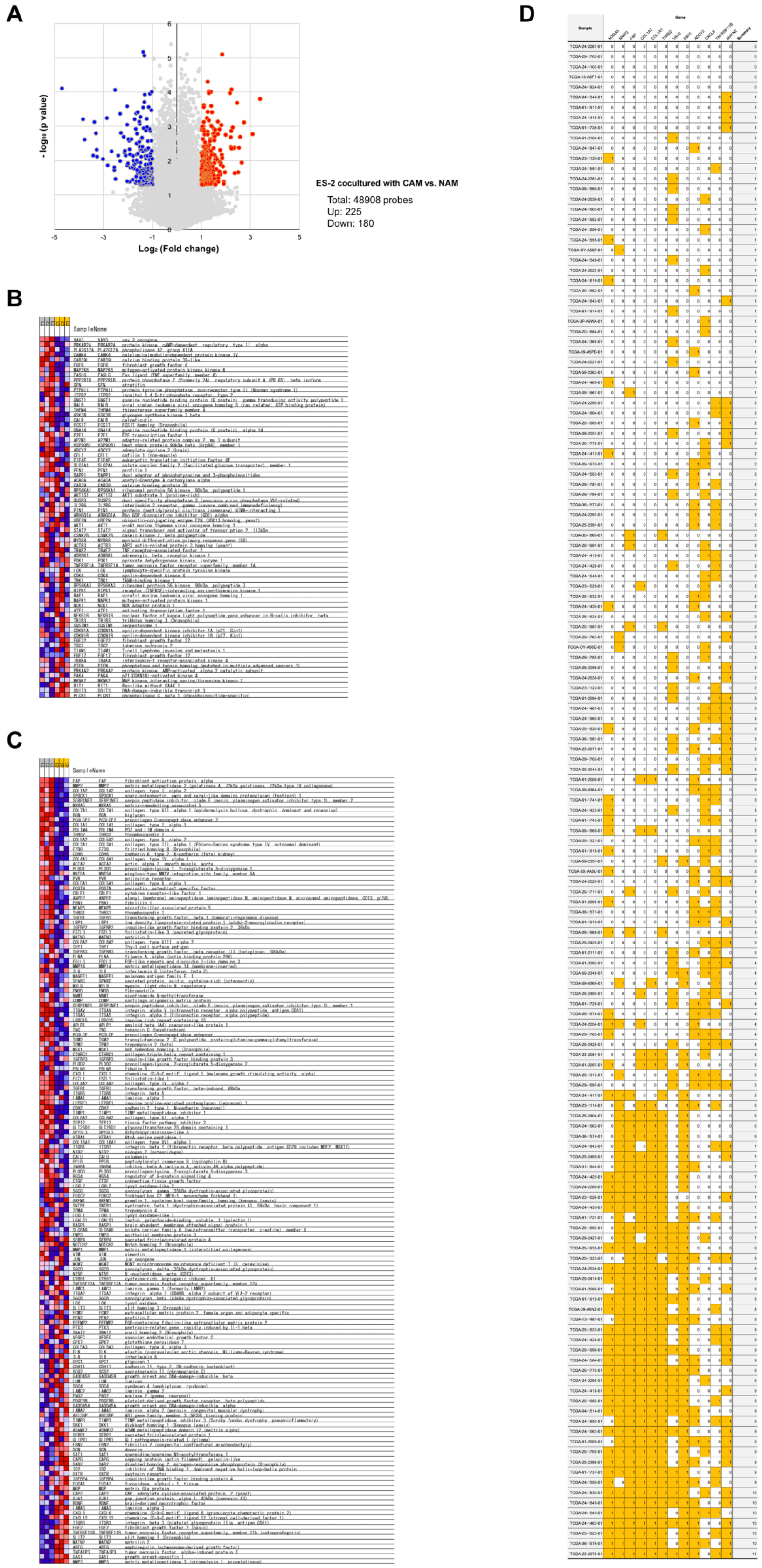

Figure S4. Gene expression analysis of ES-2 cells co-cultured with TGF-β1-treated and untreated HPMCs

A) A volcano plot illustrating the fold changes in expression and P values for all genes identified in ES-2 cells co-cultured with TGF-β1-treated HPMCs compared with those co-cultured with untreated HPMCs. B, C) Lists of Akt- and EMT-related genes identified in the GSEA. D) A complete list of patients used for the clinical analysis (n = 149) and the 12 valid genes identified in their transcriptional signature. Orange squares indicate positive identification of each gene as counting one point.

A

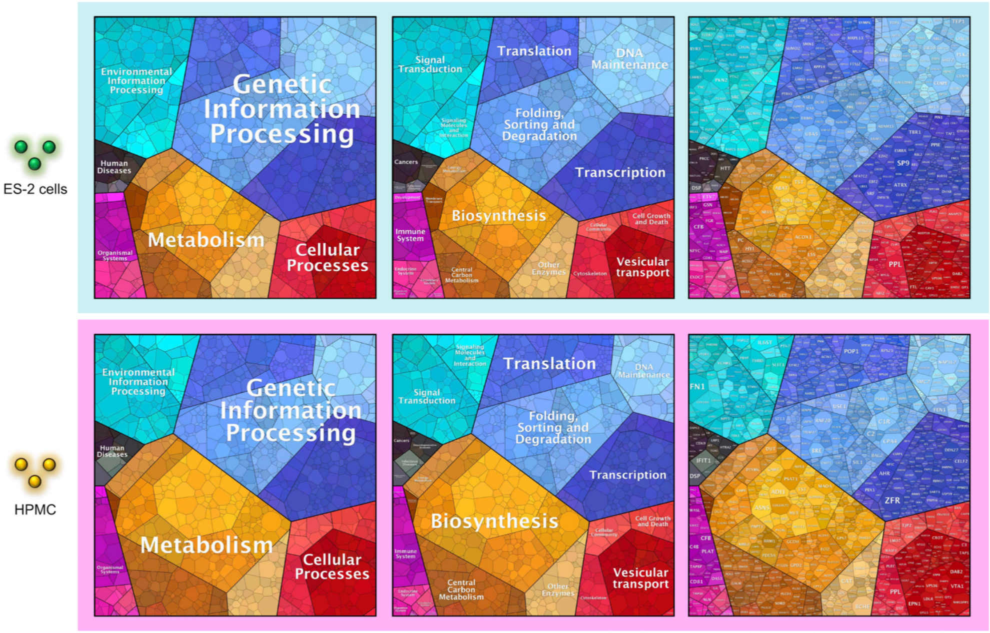

B

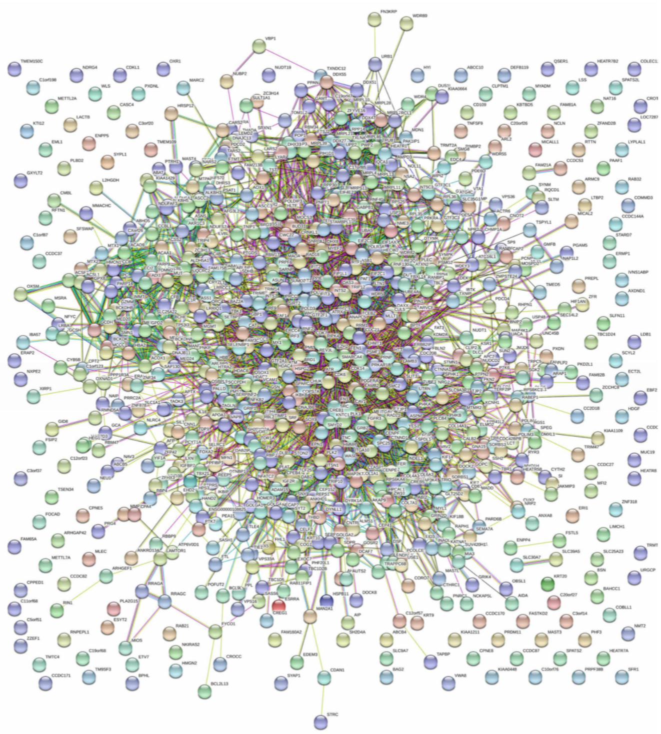

Pathway description	Gene count	FDR	Matching proteins in your network (labels)
ECM-receptor interaction	12	0.0163	COL1A1,COL1A2,COL4A3,COL6A3,FN1, HMMR,HSPG2,ITGA10,ITGAV,LAMB2, LAMB3,TNC
Viral carcinogenesis	19	0.0163	ATP6V0D1,CDK6,CREB1,CREB5,GRB2, GSN,GTF2A1,HDAC4,HDAC6,HDAC7, IL6ST,IRF3,IRF9,NFKB2,RANBP1,RBL2, RBPJ,SRC,SRF
Valine, leucine and isoleucine degradation	8	0.0259	ABAT,ACAA1,ALDH6A1,AOX1,BCKDHB, MCCC1,MUT,PCCB

Figure S5. Proteomaps and Interactome depicting the function of up-regulated FN1 on OCAMs (related to Figure 4)

A) Proteomaps of low level (left panels), middle level (middle panels), and high level (right panels), in which areas of each protein reflect the magnitude of the geographical average fold change for ES-2 cells co-cultured with TGF-β1-treated HPMCs over those cultured with untreated HPMCs, and for TGF-β1-treated HPMCs over untreated HPMCs. B) A complete interactome analysis comprised of only those proteins associated with differentially expressed proteins in both ES-2 and HPMCs cell cultures. The lower panel identifies pathways that were determined to be significantly associated with the differentially expressed proteins.

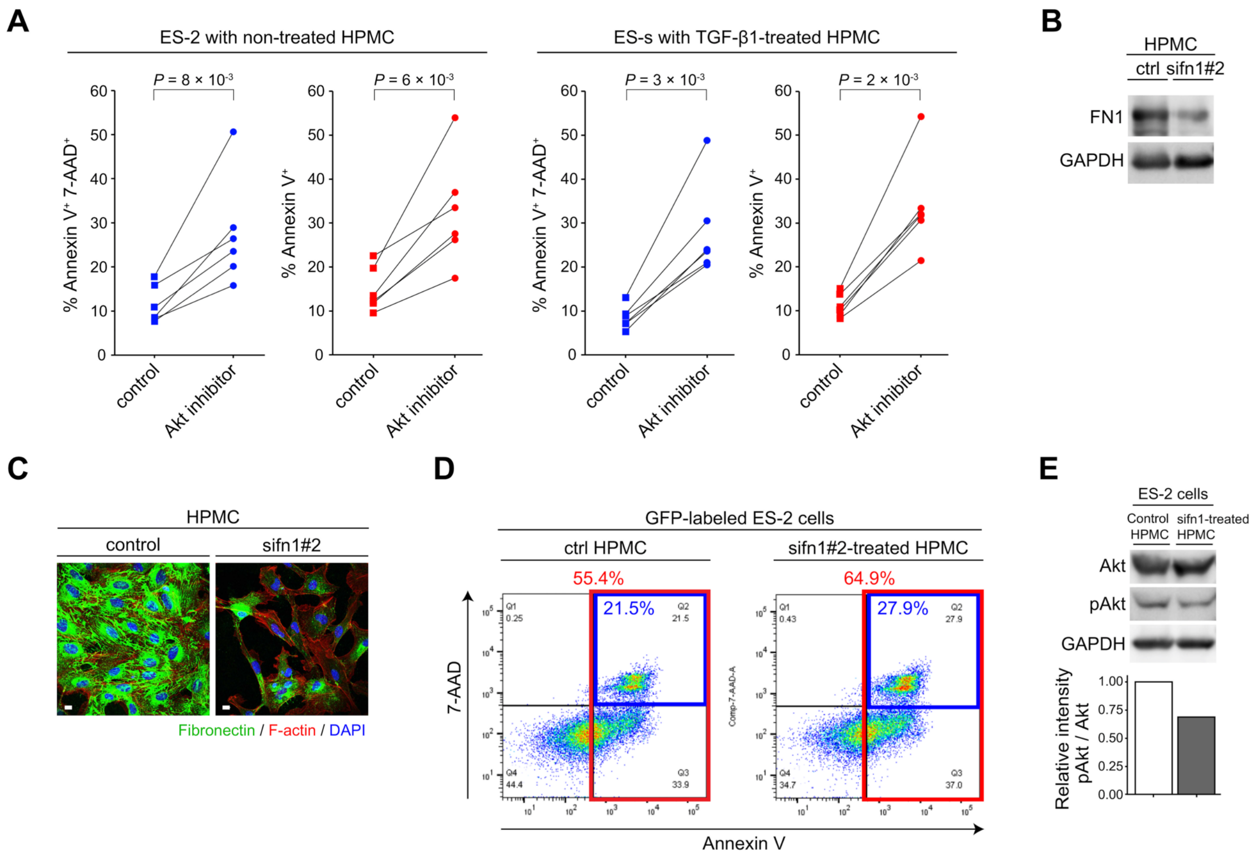

Figure S6. Activation of the FN1-induced Akt signaling in OvCa cells induces apoptosis (related to Figure 5)

A) Flow cytometric analysis of Annexin V and 7-AAD expression in GFP-labeled ES-2 cells isolated from co-cultures with HPMCs in the presence or absence of an Akt inhibitor. The proportion cells staining positive for both Annexin V and 7-AAD, or Annexin V alone are illustrated (n = 6). B, C) Immunoblot analysis and immunofluorescence of HPMCs treated with control siRNA or siFN1#2. D) Flow cytometric analysis illustrating the proportion of GFP-labeled ES-2 cells isolated from co-culture with HPMCs treated with control siRNA or siFN1#2 staining positive for Annexin V and 7-AAD. Scale bars, 10 μm. E) Immunoblot analysis of ES-2 cells co-cultured with HPMCs treated with control siRNA or siFN1#2.

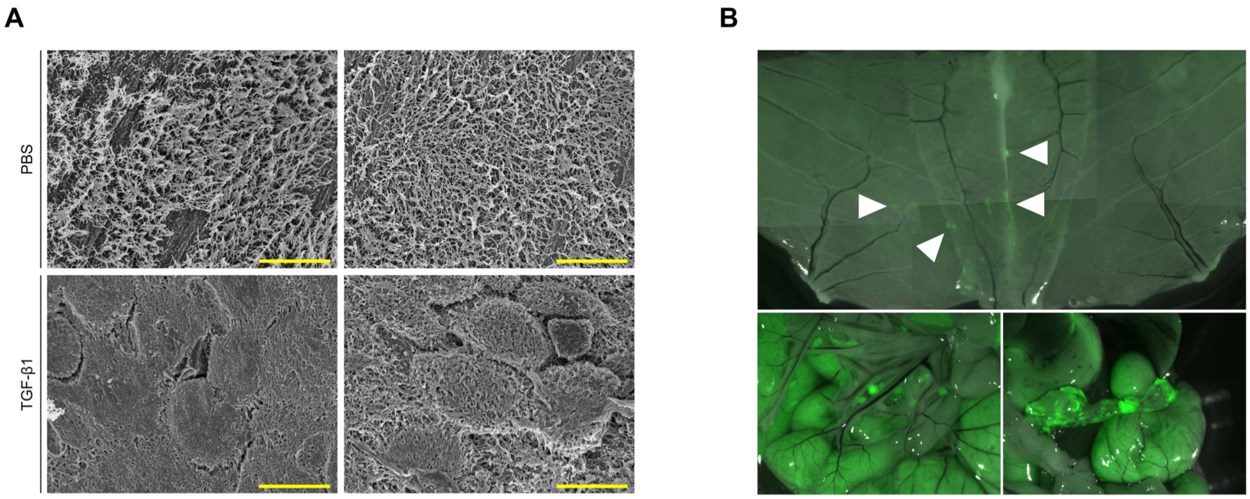

Figure S7. Activation of Akt signaling via TGF-β1 stimulation induces morphological changes to mice peritoneum

A) Images from SEM of the peritoneal surface treated with control PBS or TGF-β1. Scale bars, 10 μm. B) Stereoscopic images of the parietal (upper) and visceral peritoneum (lower) of mice. A green spot (white arrowhead) represents xenograft tumor in CMFDA-stained ES-2 cells.

SUPPLEMENTARY REFERENCES

1 Liu W, Kajiyama H, Shibata K, Koya Y, Senga T, Kikkawa F. Hematopoietic lineage cell-specific protein 1 immunoreactivity indicates an increased risk of poor overall survival in patients with ovarian carcinoma. *Oncol Lett* **2018**;15:9406–12.

2 Mitsui H, Shibata K, Mano Y, Suzuki S, Umezu T, Mizuno M, Yamamoto E, Kajiyama H, Kotani T, Senga T, Kikkawa F. The expression and characterization of endoglin in uterine leiomyosarcoma. *Clin Exp Metastasis* **2013**;30:731–40.

3 Koya Y, Liu W, Yamakita Y, Senga T, Shibata K, Yamashita M, Nawa A, Kikkawa F, Kajiyama H. Hematopoietic lineage cell-specific protein 1 (HS1), a hidden player in migration, invasion, and tumor formation, is over-expressed in ovarian carcinoma cells. *Oncotarget* **2018**;9:32609–23.

4 Tan TZ, Yang H, Ye J, Low J, Choolani M, Tan DS, Thiery JP, Huang RY. CSIOVDB: a microarray gene expression database of epithelial ovarian cancer subtype. Oncotarget **2015**;6:43843-52.
